# Postoperative bleeding after dentoalveolar surgery in patients with thrombocytopenia—are prophylactic platelet transfusions necessary?

**DOI:** 10.1007/s00520-024-08917-1

**Published:** 2024-10-07

**Authors:** Johan Lundström, Samuel Wiqvist, Martin Jädersten, Victor Tollemar, Karin Garming Legert

**Affiliations:** 1 Department of Dental Medicine, Karolinska Institutet, Stockholm, Sweden; 2 Public Dental Services, Stockholm, Sweden; 3 Department of Learning, Informatics, Management & Ethics (LIME), Karolinska Institutet, Stockholm, Sweden; 4 Department of Hematology, Karolinska University Hospital, Stockholm, Sweden; 5 Center for Hematology and Regenerative Medicine (HERM), Karolinska Institutet, Stockholm, Sweden

**Keywords:** Thrombocytopenia, Oral surgery, Postoperative bleeding, Dentoalveolar surgery, Hematology, Platelets

## Abstract

**Purpose:**

Benefits of prophylactic platelet (PLT) transfusion before dentoalveolar surgery are unclear. This study investigated the effect of prophylactic PLT transfusions on the incidence of postoperative bleeding (POB) in patients with thrombocytopenia and a PLT count ≤ 75*10^9^/L.

**Methods:**

The cohort in this retrospective study comprised 83 patients with thrombocytopenia ≤ 75*10^9^/L who had undergone dentoalveolar surgery. Exclusion criteria were other coagulation deficiencies or medications that would affect hemostasis. In all, 144 teeth had been removed. POB events were extracted and compared between the group that had received prophylactic PLT transfusion before dentoalveolar surgery and the group that had not.

**Results:**

POB events were observed in 5 of 83 patients (6.0%) who had a median PLT count of 35*10^9^/L before any transfusion. The group with no postoperative bleeding (NPOB) had a median PLT count of 34*10^9^/L. Two (4.2%) of the 48 patients who had received prophylactic PLT transfusions before dentoalveolar surgery developed POB. Three (8.6%) of the 35 patients who had not received a transfusion experienced POB. The difference between these two groups was not significant (*p* = 0.646). When two or more teeth were removed in the same session, a significantly higher incidence of POB was observed (*p* = 0.042).

**Conclusions:**

Our data indicate that prophylactic PLT transfusions in thrombocytopenic patients with PLT counts ≤ 75*10^9^/L do not reduce the incidence of POB after dentoalveolar surgery. However, caution is warranted when extracting multiple teeth in the same surgical session since we found this to be significantly associated with an increased risk of POB.

**Supplementary Information:**

The online version contains supplementary material available at 10.1007/s00520-024-08917-1.

## Introduction

Several conditions can affect the number of platelets (PLT) in the blood, including hematological malignancies and immune-mediated thrombocytopenia. PLTs are important for primary hemostasis, and a reduced PLT count could result in prolonged bleeding time at the site of injury. Thrombocytopenia is commonly defined as a PLT count of < 150*10^9^/L [1]. The U.S. Department of Health and Human Services Guiding Document “Common Terminology Criteria for Adverse Events (CTCAE)” recommends four grades of severity for thrombocytopenia [2].

Few studies have been published on the management of dentoalveolar surgery in patients with thrombocytopenia [3–5]. Varying guidelines and recommendations have been proposed internationally. Many state that prophylactic PLT transfusions should be administered before dentoalveolar surgery if patients have a PLT count of < 50*10^9^/L. This threshold has been used in dental procedures; it is an extrapolation from surgical guidelines and lacks solid scientific support [3, 4].

Because PLT transfusions are expensive and not without risk, it is important to evaluate whether they are needed and should be used before dentoalveolar surgery. Units of PLT are in high demand in hospital settings and their use should not be unnecessarily squandered.

Earlier studies and reviews in dentoalveolar surgery have observed no significant difference in the incidence of POB between patients who have received prophylactic PLT transfusions and those who have not [3, 4]. The PLT count threshold for inclusion in previous studies has generally been < 100*10^9^/L [3, 5], and medications that could affect hemostasis have not always been excluded [3].

Thus, this study retrospectively evaluated POB complications in patients with a PLT count of ≤ 75*10^9^/L prior to dentoalveolar surgery and had received no other anticoagulant medication that would affect hemostasis, including non-steroidal anti-inflammatory drugs (NSAIDs). The primary aim was to evaluate differences in POB frequency among patients who had received prophylactic PLT transfusion and those who had not. The secondary aim was to evaluate differences and independent risks between the POB group and the non-postoperative bleeding (NPOB) group regarding hematological diagnosis, dental diagnosis, CTCAE grade, type of dentoalveolar surgery, and use of local hemostatic treatment.

## Materials and methods

This retrospective study was conducted in collaboration with the Public Dental Service, the Department of Orofacial Medicine at Karolinska University Hospital; the Department of Dental Medicine, Division of Orofacial Medicine at Karolinska Institutet; and the Department of Hematology at Karolinska University Hospital.

Patients were extracted from referrals by the Hematology Department who had a primary hematological diagnosis and were in need of a dental examination prior to medical treatment between January 2010 and August 2022. Duplicate referrals and patients under 18 years of age were excluded. In all, 2334 unique patients were initially included in the study. To be included further, the patient needed to have had dental extractions or other dentoalveolar surgery, and have had a pre-surgical (within 10 days) PLT count of ≤ 75*10^9^/L. The CTCAE system for grading thrombocytopenia was used to determine the severity of thrombocytopenia in the selected patients: grade 1 was defined as a PLT count extending from the lower limits of normal to 75*10^9^/L; it is considered mild thrombocytopenia and the patient is usually asymptomatic or has only mild symptoms. Grade 2 was defined as a PLT count of < 75 down to 50*10^9^/L, which is considered moderate thrombocytopenia. Grade 3 was defined as a PLT count of < 50 down to 25*10^9^/L, which is considered severe and medically significant. Grade 4 was defined as a PLT count below 25*10^9^/L, which is considered life-threatening [2, 6]. Our study included patients with CTCAE thrombocytopenia grades 2–4. One patient with a PLT count of 75*10^9^/L was included and placed in the CTCAE grade 2 group.

Exclusion criteria were other known coagulation deficiencies and anticoagulant medications that would affect primary hemostasis, secondary hemostasis, or fibrinolysis, (including NSAIDs). Patients with a PLT count taken more than 10 days prior to dentoalveolar surgery were also excluded. A patient was regarded as having been given a prophylactic PLT transfusion if it had been administered within 24 h of surgery.

Number of teeth extracted at each visit was recorded, as was the dental diagnosis that was set before any tooth extraction or other dentoalveolar surgery. Dental diagnoses were classified as periodontitis, symptomatic/asymptomatic apical periodontitis, symptomatic/asymptomatic pulpitis and/or fracture, pericoronitis, and other (biopsy and alveoloplasty). Data on type of dentoalveolar surgery were collected in two groups: (1) non-surgical or surgical removal of one tooth (including biopsy and alveoloplasty) and (2) non-surgical and/or surgical removal of ≥ two teeth. When multiple teeth were extracted at the same session, whether teeth were adjacent to each other was noted.

Use of local hemostatic treatment was recorded as a single use or as a combination of the following: resorbable sutures (Vicryl™, Ethicon Inc., Somerville, NJ, USA), resorbable products (Spongostan™ and Surgicel™, Ethicon Inc.; or Lyostypt®, Braun Medical Inc., Bethlehem, PA, USA), and local application of tranexamic acid 100 mg/ml (Cyklokapron®, Pfizer Inc., New York, NY, USA).

POB events were recorded and defined as difficulties achieving primary hemostasis perioperative or if the patient contacted an Orofacial Medicine Department, a Public Dental Service Emergency Clinic, or a Hematology Department within 48 h after surgery to report unexpected bleeding complications and was in need of a clinical intervention.

**Fig. 1:**
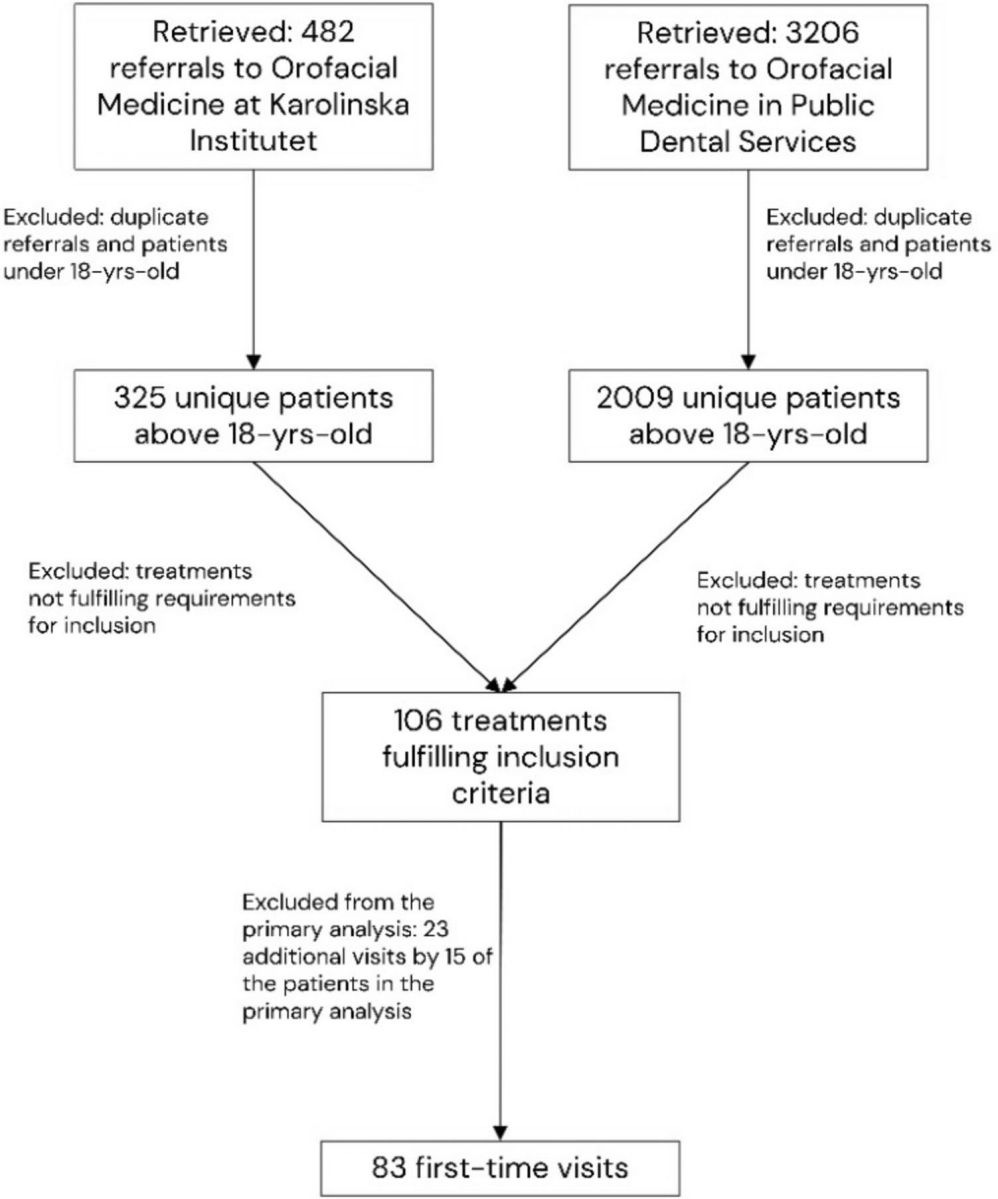
Flow chart of patient selection from the retrieved clinical charts (*n* = 2,334); the primary analysis included 83 patients with first-time visits. The secondary analysis included 15 patients who had additional treatments (*n* = 23 treatments not including the first visit)

Eighty-three patients fulfilled these criteria and were included in the study. Fifteen patients had additional visits where dentoalveolar surgery was performed and fulfilled these criteria more than once, adding up to a total of 23 additional visits. Due to the complex patient and time-domain dependencies and the small size of the dataset, which add complexity to modeling, the primary analysis only included 83 first-time visits (Fig. 1). The secondary analysis included all visits (*n* = 106; Fig. 1).

### Statistical analysis

In the primary analysis, categorical variables concerning POB/NPOB were described as count and percentages, correspondingly numerical variables were described as medians with their ranges. We used Fisher’s exact test and Wilcoxon rank-sum test to test for differences in categorical and numerical variables in relation to POB/NPOB, respectively.

Besides describing the variables and testing for differences in relation to POB/NPOB, we also calculated effect sizes and their confidence intervals. For interpreting the magnitudes of the effects, we employed the following commonly used thresholds: Cohen’s h: small > 0.2, medium > 0.5, and large > 0.8 [7]. For Cramer’s V: small > 0.1, medium > 0.3, and large > 0.5 [7]. For Cohen’s d: small > 0.2, medium > 0.5, and large > 0.8 [7].

In an analogous manner, we analyzed pre-transfusion PLT counts and POB events in relation to transfusion/no transfusion.

In the secondary analysis, we used a mixed logistic regression model to model POB incidence for all 106 visits (83 first and 23 follow-up visits). The covariates of interest were included as fixed effects. The patient-specific risk was modelled using a random intercept term for each patient. To account for the time dependency of the follow-up visits, we employed a temporal inter-group covariate structure. The model fit was evaluated using Nakagawa’s R2 [8] and the area under the receiver operator curve [9].

Statistical analyses were carried out in R v4.4.0. The code for our use of R is available in “Online Resource 1”.

## Results

### Cohort

Table 1 presents the characteristics of the 83 patients at their first visits. “Online Resource 2” summarizes patient characteristics at the 23 additional visits. In the primary analysis, PLT count was measured a mean of 1.1 days (range 0–9) prior to dentoalveolar surgery. Forty-eight patients received prophylactic PLT transfusion prior to treatment (mean 1.5 units PLT, range: 1–4). The units were administered a mean of 4.0 h (range 1–24) before dentoalveolar surgery. Only 9 patients had a new PLT count taken after prophylactic PLT transfusion with a mean PLT count increase of 33*10^9^/L (range -2 to 68*10^9^/L). The PLT count in 1 patient had decreased from 11*10^9^/L to 9*10^9^/L. Thirty-five patients had received no prophylactic PLT transfusion.

**Table 1:** Patient characteristics on the first visit (*n* = 83 patients). Categorical variables are reported with counts (percentages), and numerical variables are reported with medians (ranges). All results are reported with a precision of one decimal

Patient characteristics	
Gender, n (%)	
Male	57 (68.7)
Female	26 (31.3)
Age (years), median (range)	65 (24, 85)
PLT count before any transfusion (*10^9^/L), median (range)	34 (5, 75)
Hematological diagnosis, n (%)	
MDS/AML	65 (78.3)
Myeloma	4 (4.8)
Lymphoma^a^	8 (9.6)
Other^b^	6 (7.2)
CTCAE, thrombocytopenia severity (*10^9^/L), n (%)	
Grade 2 (PLT count 75–50)	22 (26.5)
Grade 3 (PLT count 49–25)	34 (41)
Grade 4 (PLT count 24–)	27 (32.5)
PLT transfusion ≤ 24 h, n (%)	
Yes	48 (57.8)
No	35 (42.2)
Local hemostatic treatment, n (%)	
Yes	71 (85.5)
No	12 (14.5)
No. of teeth extracted, median (range)	1 (0, 7)
Extraction diagnosis, n (%)	
Periodontitis	31 (37.3)
Symptomatic/asymptomatic apical periodontitis	33 (39.8)
Symptomatic/asymptomatic pulpitis/fracture	16 (19.3)
Pericoronitis	1 (1.2)
Other (biopsy, alveoplasty)	2 (2.4)
Type of dentoalveolar surgery, n (%)	
Non-surgical/surgical extraction 1 tooth (also: biopsy, alveoplasty)	55 (66.3)
Non-surgical/surgical extraction ≥ 2 teeth	28 (33.7)
Adjacent teeth ≥ 2, n (%)	
Yes	19 (22.9)
No	64 (77.1)

*PLT* platelet, *AML/MDS* acute myeloid leukemia/myelodysplastic syndrome, *CTCAE* Common Terminology Criteria for Adverse Events

^a^including acute lymphoblastic leukemia, chronic lymphocytic leukemia, mantle cell lymphoma, and diffuse large B cell lymphoma

^b^including hemophagocytic lymphohistiocytosis and aplastic anemia

### Postoperative bleeding

In all, 144 teeth were removed, one biopsy was done, and one alveoplasty performed. The biopsy and alveoplasty were considered similar in scope to a non-surgical or surgical extraction of one tooth and, thus, were placed in the same group. Five (6.0%) of the 83 patients experienced POB events after dentoalveolar surgery. Two of the 5 received PLT transfusions to control the bleeding as well as compression and local hemostatic treatment. The other 3 only required compression and local hemostatic treatment.

Of these five POB events, all were males and two patients had received prophylactic PLT transfusion within 24 h before their dentoalveolar surgery and three had not (Table 2).

**Table 2:** Baseline characteristics of the patients who did or did not experience post-operative bleeding

Variable	POB (*n* = 5)	NPOB (*n* = 78)	*p*-value	Effect size est (confidence interval)
Gender, n (%)				
Male	5 (100)	52 (66.7)	0.319	1.2 (0.3, 2.1)
Female	0 (0)	26 (33.3)		
Age (years), median (range)	64 (42,78)	65.5 (24, 85)	0.916	0.1 (-0.8, 1)
PLT count before any transfusion (*10^9^/L), median (range)	35 (13, 62)	33.5 (5, 75)	0.781	0.1 (-0.8, 1)
Hematological diagnosis, n (%)				
MDS/AML	4 (80)	61 (78.2)	0.389	0.2 (0, 0.4)
Myeloma	1 (20)	3 (3.8)		
Lymphoma^a^	0 (0)	8 (10.3)		
Other^b^	0 (0)	6 (7.7)		
CTCAE, thrombocytopenia severity (*10^9^/L), n (%)				
Grade 2 (PLT count 75–50)	2 (40)	20 (25.6)	0.608	0.1 (0, 0.3)
Grade 3 (PLT count 49–25)	1 (20)	33 (42.3)		
Grade 4 (PLT count 24–)	2 (40)	25 (32.1)		
PLT transfusion ≤ 24 h, n (%)				
Yes	2 (40)	46 (59)	0.646	-0.4 (-1.3, 0.5)
No	3 (60)	32 (41)		
Local hemostatic treatment, n (%)				
Yes	5 (100)	66 (84.6)	1	0.8 (-0.1, 1.7)
No	0 (0)	12 (15.4)		
No. of teeth extracted, median (range)	2 (1, 3)	1 (0, 7)	0.069	0.4 (-0.5, 1.3)
Extraction diagnosis, n (%)				
Periodontitis	1 (20)	30 (38.5)	0.062	0.5 (0.3, 0.7)
Symptomatic/asymptomatic apical periodontitis	3 (60)	30 (38.5)		
Symptomatic/asymptomatic pulpitis/fracture	0 (0)	16 (20.5)		
Pericoronitis	1 (20)	0 (0)		
Other (biopsy, alveoplasty)	0 (0)	2 (2.6)		
Type of dentoalveolar surgery, n (%)				
Non-surgical/surgical extraction 1 tooth (also: biopsy alveoplasty)	1 (20)	54 (69.2)	0.042*	-1 (-1.9, -0.1)
Non-surgical/surgical extraction ≥ 2 teeth	4 (80)	24 (30.8)		
Adjacent teeth ≥ 2, n (%)				
Yes	2 (40)	17 (21.8)	0.322	0.4 (-0.5, 1.3)
No	3 (60)	61 (78.2)		

*POB* postoperative bleeding, *NPOB* no postoperative bleeding, *PLT* platelet, *AML/MDS* acute myeloid leukemia, myelodysplastic syndrome, *CTCAE* Common Terminology Criteria for Adverse Events

^a^including acute lymphoblastic leukemia, chronic lymphocytic leukemia, mantle cell lymphoma, and diffuse large B cell lymphoma

^b^including hemophagocytic lymphohistiocytosis and aplastic anemia

Effect size: Binary: Cohen's* h*; categorical (> 2 levels): Cramer’s; numerical: Cohen's *d*; *p < 0.05

Two POB events occurred in the CTCAE grade 2 group (*n* = 22). Neither of the two patients had received any prophylactic PLT transfusions, and the mean PLT count was 61*10^9^/L. One POB event occurred in the CTCAE grade 3 group (*n* = 34): the patient had received prophylactic PLT transfusion before surgery and had a PLT count of 35*10^9^/L before transfusion. Two POB events occurred in the CTCAE grade 4 group (*n* = 27). One of these two patients had received prophylactic PLT transfusion and one had not. The mean pre-transfusion PLT count for these two was 16*10^9^/L (Table 2).

A median of 2.0 (range 1–3) teeth in the POB group and 1.0 (range 0–7) in the NPOB group had been extracted. A significantly higher incidence of POB occurred when ≥ 2 teeth were removed non-surgically and/or surgically compared with when only one tooth was removed non-surgically or surgically (*p* = 0.042; Table 2).

### Prophylactic PLT transfusion and thrombocytopenia

There was no significant difference regarding the incidence of POB with respect to which CTCAE group the patient belonged to prior to transfusion (*p* = 0.608; Table 2). The incidence of POB events between those patients who had received a prophylactic PLT transfusion within 24 h of surgery and those who had not was not significant (*p* = 0.646; Table 3). Prior to any transfusion, median PLT count in the POB group was 35*10^9^/L; for the NPOB group, it was lower: 34*10^9^/L. The use of local haemostatic treatment did not differ between the POB and NPOB groups (*p* = 1.000). The dental diagnosis for tooth extraction or dentoalveolar surgery was not significantly related to the incidence of POB (*p* = 0.062).

**Table 3:** Bleeding events and PLT count before any transfusion regarding transfusion/no transfusion

Variable	Transfusion (*n* = 48)	No transfusion (*n* = 35)	*p*-value	Effect size est (confidence interval)
PLT count before any transfusion (*10^9^/L), median (range)	26.5 (5,72)	48 (9,75)	1.72e-05***	-1.1 (-1.6, -0.6)
Bleeding events, n (%)				
POB	2 (4.2)	3 (8.6)	0.646	-0.2 (-0.6, 0.3)
NPOB	46 (95.8)	32 (91.4)		

*PLT* platelet, *POB* postoperative bleeding, *NPOB* no postoperative bleeding

Effect size: Binary: Cohen's* h*; numerical: Cohen's *d*; ***p < 0.001

In the secondary analysis, which included 23 additional visits along with the 83 first time visits (thus, *n* = 106 visits), seven POB events occurred (6.6%). Also in the secondary analysis, receiving or not receiving a prophylactic PLT transfusion within 24 h of surgery was not significantly related to the incidence of POB (*p* = 0.52). However, a significant relationship was found between an increased incidence of POB events (*p* = 0.033) and extraction of ≥ 2 teeth non-surgically or surgically in the same session (Table 4).

**Table 4:** Secondary analysis of all visits (first visit and additional visits) using three mixed regression models with robust standard errors. The estimated coefficient, standard error, and *p*-value of the coefficient of the fixed effect summarize the models. The baseline cases for the models are CTCAE 2, PLT transfusion ≤ 24 h: No, and non-surgical/surgical extraction of 1 tooth including biopsy and alveoplasty

Model	Fixed Effect	Regression analysis		
		Estimated coefficient	SE	*p*-value
1	CTCAE grade 3	-0.6	1	0.578
	CTCAE grade 4	0.2	0.9	0.848
2	PLT transfusion ≤ 24 h: Yes	-0.5	0.8	0.52
3	Dentoalveolar surgery: non-surgical/surgical extraction ≥ 2 teeth	2.4	1.1	0.033*

*SE* standard error, *CTCAE* Common Terminology Criteria for Adverse Events – severity of thrombocytopenia, *PLT* platelet

^*^p < 0.05

**Fig. 2:**
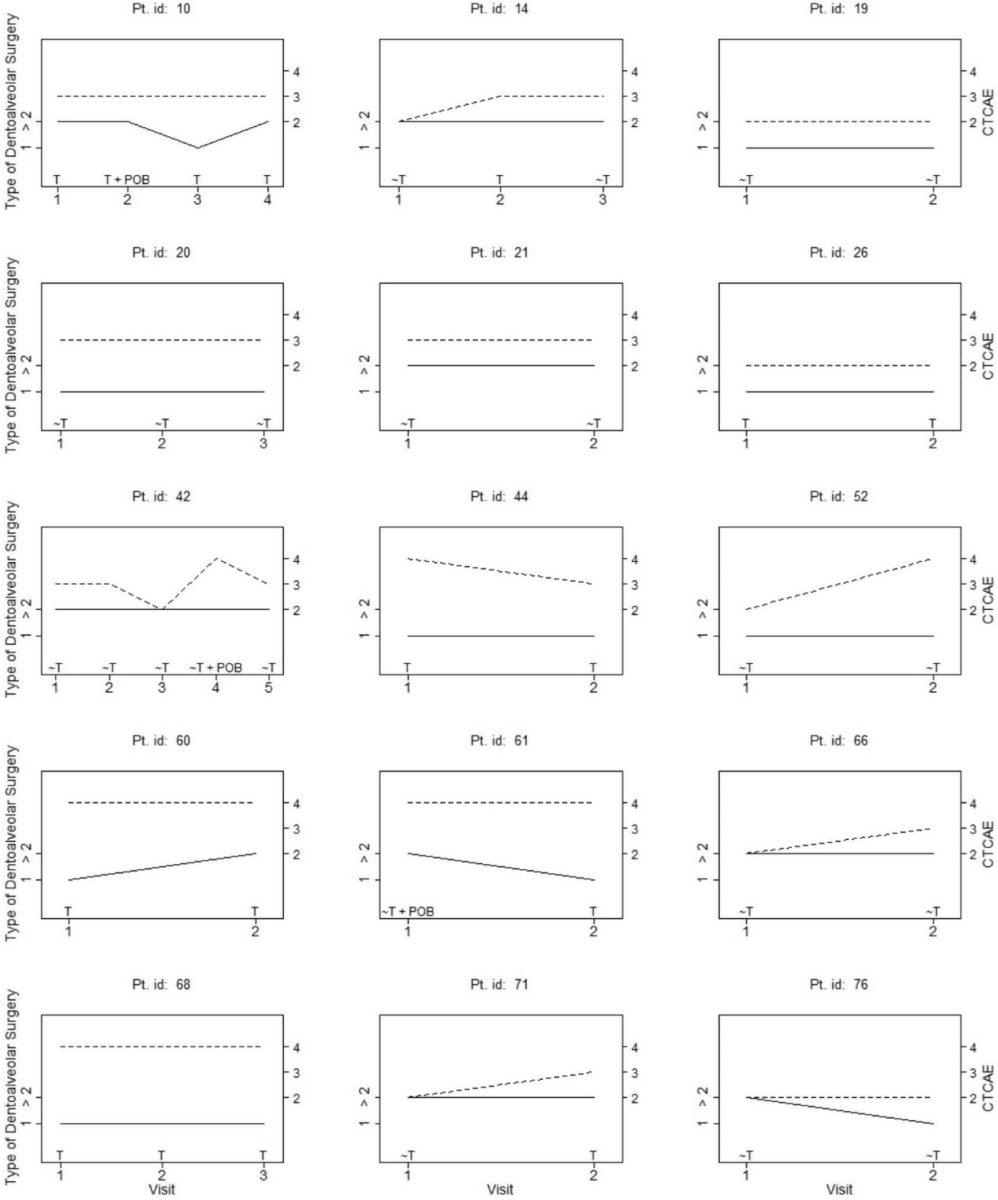
Time-based dependencies (type of dentoalveolar surgery, CTCAE 2–4 and transfusion/no transfusion) associated with postoperative bleeding events. 15 patients with first visits and a total of 23 additional visits are included. Solid lines indicate the type of dentoalveolar surgery, on the left axis. Dotted lines indicate CTCAE group, on the right axis. ***CTCAE*** Common Terminology Criteria for Adverse Events – severity of thrombocytopenia, ***T*** transfusion, ~ ***T*** no transfusion, ***POB*** postoperative bleeding, ***1*** non-surgical/surgical extraction 1 tooth incl. biopsy and alveoplasty, > ***2*** non-surgical/surgical extraction ≥ 2 teeth, ***Pt***. Patient

Figure 2 illustrates the time-based dependencies (type of dentoalveolar surgery, CTCAE grade, and transfusion/no transfusion) at each treatment and the association with POB events for the 15 patients with multiple visits (*n* = 23 additional visits).

## Discussion

To our knowledge, this study is one of the largest retrospective studies conducted with patients undergoing dentoalveolar surgery and having a relatively severe thrombocytopenia (*n* = 83; ≤ 75*10^9^/L); no known coagulation deficiencies; and ingestion of no known anticoagulant medication that would affect primary hemostasis, secondary hemostasis, or fibrinolysis, including NSAIDs. Local hemostatic treatment did not significantly differ between POB or NPOB group, only 12 of the 83 patients received no local hemostatic treatment. In line with previous studies and reviews [3–5], we found no significant support that the administration of prophylactic PLT transfusions would reduce the incidence of POB in patients with thrombocytopenia. We hypothesized that a tooth with periodontal disease would increase the incidence of POB due to granulation tissue; however, the present study found no significant relation between dental diagnosis and POB events. Our data, however, did show a significantly higher risk of POB when two or more teeth were removed, non-surgically and/or surgically, in the same session.

In the present study, the general incidence of POB in patients with thrombocytopenia and a CTCAE grade 2–4 was 6.0%. Few studies have evaluated the incidence of POB in healthy patients after dentoalveolar surgery. One multi-center study reported an 2.1% incidence of POB in a group of healthy controls [10]. Kang and colleagues recently investigated POB events in 220 patients undergoing dentoalveolar surgery, where 130 patients had a PLT count of < 150*10^9^/L and 90 patients had a PLT count of 150*10^9^/L – 250*10^9^/L. POB occurred in 7.7% of the patients with thrombocytopenia and 1.1% in patients with a normal PLT count [11], which are in line with the findings of our study.

Karasneh et al. found no evidence in their systematic review to support the long-standing PLT count threshold of < 50*10^9^/L for administration of prophylactic PLT transfusion prior to dentoalveolar surgery [4]. The 2018 clinical practice guidelines of the American Society of Clinical Oncology (ASCO) recommended a threshold of 40–50*10^9^/L for prophylactic PLT transfusion before major invasive surgery on patients with no other associated coagulation abnormalities [12]. For bone marrow aspirations, bone marrow biopsies, and the removal of central venous catheters, ASCO recommendations state that patients with a PLT count of < 20*10^9^/L do not require prophylactic PLT transfusions [12].

One unit of PLT costs approximately USD 528 [13] in Sweden and up to USD 4,436 in the US. [14]. Over the course of our study (12 years), our cohort had received 86 units of PLT, at a cost of roughly USD 43,000 in Sweden and USD 382,000 in the US. Our data demonstrated no significant reduction in the incidence of POB when prophylactic platelet transfusions were administered prior to dentoalveolar surgery. The lack of benefit in this setting, in combination with the shortage of blood components and the high costs associated with transfusion, argue for a more restrictive PLT transfusion strategy compared with current recommendations in several international guidelines.

The clinical outcome of PLT transfusion is also unpredictable. The recent Karlström et al. study on 40 patients with severe thrombocytopenia reported the effect of PLT transfusion by measuring PLT count at three time points: before transfusion, 1 h after transfusion, and 18–24 h after transfusion. One hour after transfusion, the mean PLT count increment was 14.6*10^9^/L and after 18–24 h, 7.1*10^9^/L. Interestingly, this study showed that the increment in PLT count was less than 7.5*10^9^/L in over one-third (35%) of the patients [15]. In our study, only 18.8% of the transfused patients had a post-transfusion PLT count taken so the effect on PLT count cannot be robustly evaluated.

More importantly, PLT transfusions are not risk free. Although they account for almost 10% of all transfused blood products, they are responsible for more than 25% of reported adverse events [16]. Acute reactions due to PLT transfusion may include febrile non-hemolytic transfusion reactions, allergic reactions, transfusion-associated sepsis, transfusion-associated lung injury (TRALI), and post-procedure thrombosis [17, 18]. One prospective observational study on 376 patients given PLT transfusions identified 19 thrombotic events (5%) within 7 days following transfusion. This is about a four times higher rate than what would be expected in patients with a cancer diagnosis [18]. The large, retrospective study of Khorana et al. reported that transfusions with red blood cells and with PLT were both associated with increased risks of venous and arterial thrombotic events, as well as mortality, in hospitalized patients with a cancer diagnosis [19]. TRALI is an acute respiratory syndrome with a complex etiology and one of the leading causes of transfusion-related deaths [6].

Our primary analysis found only 5 POB events in the 83 patients, and none of the POB events were life-threating. Only 2 required PLT transfusions following the events, which according to a recent review is questionable to have any effect [20]. Three of the POB events were easily managed by local compression and local hemostatic treatment which seems to be the most important intervention [20].

Incorporating the 23 additional visits into the statistical analysis was a challenging problem since the additional visits introduced time-based dependencies. It was also reasonable to assume that each patient had their own POB risk profile that should be considered. Thus, to analyze all 106 visits together, we needed to consider the time and patient level dependencies involved. Since most patients had only made one visit (i.e., the first-time visit), the challenge was to incorporate these dependencies correctly in a model. Hence, our primary analysis excluded the 23 additional visits. Because two POB events did occur in the additional visits, we decided to analyze all 106 visits in a secondary analysis using mixed-effects regression models.

The secondary analysis agreed with the primary analysis concerning the relation of POB incidence with CTCAE grade, prophylactic PLT transfusion or no transfusion, and type of dentoalveolar surgery. The best fit of the three investigated models was the model using dentoalveolar surgery as the fixed effect, which is consistent with the finding that type of dentoalveolar surgery is the only significant modelling variable for the POB/NPOB strata. A more detailed description of the patients with POB events and their visits can be found in “Online Resource 3”.

Dentoalveolar surgery is performed in an easily accessible region of the mouth and can be easily compressed in cases of bleeding. Our study indicates that prophylactic PLT transfusions are non-significant for the incidence of POB in patients where thrombocytopenia CTCAE grade 2–4 is the only existing variable to affect hemostasis. Taking account of transfusion-related adverse effects and the general shortage of PLT transfusions in hospital settings, we argue that the long-standing recommendation of prophylactic PLT transfusions prior to dentoalveolar surgery when the patient’s PLT count is < 50*10^9^/L should be revaluated.

It is difficult to assess the risk of POB in dentoalveolar surgery compared with other invasive treatments but as mentioned, ASCO suggests that minor surgical procedures can be performed at a PLT count < 20*10^9^/L without any prophylactic PLT transfusion needed [12]. A threshold similar to this should be possible to consider for dentoalveolar surgery as well, particularly when only one tooth is removed, non-surgically or surgically, during the visit. Our study is limited by the small dataset (*n* = 83) and larger randomized, controlled trials would be needed in order to formally evaluate indications for the use of prophylactic PLT transfusions before dentoalveolar surgery.

In conclusion, no significant support was found for the idea that prophylactic PLT transfusion before dentoalveolar surgery would reduce the incidence of POB. Likewise, no association between POB incidence and CTCAE grades 2–4 was found. The only significant relation to POB events was removal of two or more teeth non-surgically and/or surgically in the same session, which warrants further investigation in prospective studies.

## Supplementary Information

Below is the link to the electronic supplementary material.

Supplementary file1 (PDF 168 KB)

Supplementary file2 (DOCX 2331 KB)

Supplementary file3 (DOCX 2278 KB)

## Data Availability

Data is provided within the manuscript or supplementary information files.
