# Supplementary material for: Postoperative bleeding after dentoalveolar surgery in patients with thrombocytopenia—are prophylactic platelet transfusions necessary?
Source: Support Care Cancer. 2024 Oct 7;32(10):703. doi: 10.1007/s00520-024-08917-1 (PMC11456549; doi:10.1007/s00520-024-08917-1)
Supplement: Supplementary file 1 — Supplementary file1 (PDF 168 KB) [file 520_2024_8917_MOESM1_ESM.pdf]

Postoperative bleeding after dentoalveolar surgery in patients with thrombocytopenia - are prophylactic platelet transfusions necessary?

Supplementary materials: Statistical Analysis

Samuel Wiqvist*

9/19/24

Table of contents

Set up 2

Functions 4

Load data 9

Analyses 11

Table 1/5 (Patient characteristics) 11
- Var: Sex 11
- Var: Age 11
- Var: Platelet count prior any transfusion 12
- Var: Hematological diagnosis 13
- Var: CTCAE 13
- Var: Platelet transfusion 14
- Var: Local hemostatic treatment 14
- Var: Nbr teeth 14
- Var: Extraction diagnosis 15
- Var: Type of dentalveolar surgery 16
- Var: Adjacent teeth 16

Table 2 (Characteristics of base line and clinical variables with respect to the strata POB/NPOB) 16
- Select pop for analyses 16
- POB ~ Sex 17
- POB ~ Age 18
- POB ~ Platelet count prior any transfusion 20
- POB ~ Hematological diagnosis 21
- POB ~ CTCAE 22
- POB ~ Platelet transfusion 23
- POB ~ Local hemostatic treatment 24
- POB ~ Nbr teeth 25
- POB ~ Extraction diagnosis 27
- POB ~ Type of dentalveolar surgery 28
- POB ~ Adjacent teeth 29

Table 3 (Characteristics of the variables post operative bleeding and PLT count before any transfusion with respect to the strata transfusion/no transfusion) 30

*Department of Learning, Informatics, Management & Ethics (LIME), Karolinska Institutet, Stockholm, Sweden. Mail: samuel.wiqvist@ki.se

Risk of POB transfusion/no transfusion . . . 30
Platelet count prior any transfusion . . . 31
Fig 2 (paths follow-up visits) . . . 33
Table 4 (Model w all obs) . . . 37
Mixed GLM with Temporal Correlation . . . 37
(Naive) Robust GLM (NB not included) . . . 44

How to Generated pdf **46**

Set up

```
setwd("P:/C7_Medstat/Enskild personals egna mappar/Samuel/Johan Lundström")

library("readxl")
library("dplyr")
```

```
Attaching package: 'dplyr'

The following objects are masked from 'package:stats':

    filter, lag

The following objects are masked from 'package:base':

    intersect, setdiff, setequal, union
```

```
library("confintr")
library("psych")
library("MASS")
```

```
Attaching package: 'MASS'

The following object is masked from 'package:dplyr':

    select
```

```
library("nlme")
```

```
Attaching package: 'nlme'

The following object is masked from 'package:dplyr':

    collapse
```

```
library("sandwich")
library("miceadds")
```

```
Loading required package: mice

Attaching package: 'mice'

The following object is masked from 'package:stats':

    filter

The following objects are masked from 'package:base':

    cbind, rbind
```

```
* miceadds 3.17-44 (2024-01-08 19:08:24)
```

```r
library("pROC")
```

```
Type 'citation("pROC")' for a citation.

Attaching package: 'pROC'

The following objects are masked from 'package:stats':

    cov, smooth, var
```

```r
library("performance")
library("lme4ord") # installed from source
```

```
Loading required package: lme4

Loading required package: Matrix

Attaching package: 'lme4'

The following object is masked from 'package:nlme':

    lmList
```

```r
library("glmmTMB")
```

```
Attaching package: 'glmmTMB'

The following objects are masked from 'package:lme4ord':

    noSpecials, splitForm
```

```r
library("rstatix")
```

```
Attaching package: 'rstatix'

The following object is masked from 'package:MASS':

    select

The following object is masked from 'package:stats':

    filter
```

```r
# print session info
writeLines(capture.output(sessionInfo()))
```

```
R version 4.4.0 (2024-04-24 ucrt)
Platform: x86_64-w64-mingw32/x64
Running under: Windows 10 x64 (build 19045)

Matrix products: default


locale:
[1] LC_COLLATE=Swedish_Sweden.utf8  LC_CTYPE=Swedish_Sweden.utf8
[3] LC_MONETARY=Swedish_Sweden.utf8 LC_NUMERIC=C
[5] LC_TIME=Swedish_Sweden.utf8
```

```
time zone: Europe/Stockholm
tzcode source: internal

attached base packages:
[1] stats     graphics  grDevices utils     datasets  methods   base

other attached packages:
 [1] rstatix_0.7.2      glmmTMB_1.1.9      lme4ord_0.0-3      lme4_1.1-35.3
 [5] Matrix_1.7-0       performance_0.11.0 pROC_1.18.5        miceadds_3.17-44
 [9] mice_3.16.0        sandwich_3.1-0     nlme_3.1-164       MASS_7.3-60.2
[13] psych_2.4.3        confintr_1.0.2     dplyr_1.1.4        readxl_1.4.3

loaded via a namespace (and not attached):
 [1] tidyselect_1.2.1    fastmap_1.1.1       TH.data_1.1-2
 [4] digest_0.6.35       rpart_4.1.23        estimability_1.5
 [7] lifecycle_1.0.4     cluster_2.1.6       survival_3.5-8
[10] magrittr_2.0.3      compiler_4.4.0      rlang_1.1.3
[13] tools_4.4.0         utf8_1.2.4          yaml_2.3.8
[16] knitr_1.46          mnormt_2.1.1        plyr_1.8.9
[19] multcomp_1.4-25     abind_1.4-5         purrr_1.0.2
[22] numDeriv_2016.8-1.1 nnet_7.3-19         grid_4.4.0
[25] fansi_1.0.6         jomo_2.7-6          xtable_1.8-4
[28] emmeans_1.10.1      iterators_1.0.14    insight_0.19.11
[31] cli_3.6.2           mvtnorm_1.2-4       rmarkdown_2.26
[34] vegan_2.6-6         generics_0.1.3      rstudioapi_0.16.0
[37] reshape2_1.4.4      minqa_1.2.6         DBI_1.2.2
[40] ape_5.8             stringr_1.5.1       splines_4.4.0
[43] parallel_4.4.0      cellranger_1.1.0    mitools_2.4
[46] vctrs_0.6.5         boot_1.3-30         glmnet_4.1-8
[49] jsonlite_1.8.8      carData_3.0-5       car_3.1-2
[52] mitml_0.4-5         foreach_1.5.2       tidyr_1.3.1
[55] glue_1.7.0          nloptr_2.0.3        pan_1.9
[58] codetools_0.2-20    stringi_1.8.3       shape_1.4.6.1
[61] tibble_3.2.1        pillar_1.9.0        htmltools_0.5.8.1
[64] R6_2.5.1            TMB_1.9.11          evaluate_0.23
[67] lattice_0.22-6      backports_1.4.1     broom_1.0.5
[70] Rcpp_1.0.12         coda_0.19-4.1       permute_0.9-7
[73] mgcv_1.9-1          xfun_0.43           zoo_1.8-12
[76] pkgconfig_2.0.3
```

Functions

```
report_num <- function(indep, dep, digits = 1) {
  #' Calcs descriptive stats for a numeical variable
  #'
  #'
  #' @param indep The indepedent continuous variable
  #' @param dep The dependent variable (here == outcome_reveresed)
  #' @param digits Nbr of digits to show prints/outputs (default = 2)

  print("Numeical Variable - Reporting")


  print("median:")
  print(round(by(indep, dep, median), digits))
```

```
  print("range")
  print(by(indep, dep, range))


  boxplot(indep ~ dep)

}
test_num <- function(indep, dep, digits = 1) {
  #' Run significant tests for a numeical variable
  #'
  #' We use the following conditions for which test to run:
  #' If skewness in any group > 1 -> Wilcoxon rank sum test
  #' If skewness in all groups < 1 and  1/2 < sd ratio < 2 -> t-test with equal variance
  #' If skewness in all groups < 1 and not  1/2 < sd ratio < 2 -> Welch's t-test
  #'
  #' @param indep The indepedent continuous variable
  #' @param dep The dependent variable (here == outcome_reveresed)
  #' @param digits Nbr of digits to show prints/outputs (default = 2)

  print("Numeical Variable - Testing")

  abs_skewes = abs(by(indep, dep, confintr::skewness))

  skewes_cond = abs_skewes >= 1

  sds = by(indep, dep, sd)

  sd_cond = unname(sds[1]/sds[2]) > 1/2 & unname(sds[1]/sds[2]) < 2

  print(sum(skewes_cond) == TRUE)
  print(is.na(sum(skewes_cond)) == TRUE)
  print(sd_cond)

  test = wilcox.test(indep  ~ dep)

  # simplify so that we only run wilcox.test
  #if (is.na(sum(skewes_cond)) == TRUE) {
  #  test = wilcox.test(indep  ~ dep)
  #} else {
  #  if (sum(skewes_cond) == TRUE) {
  #    test = wilcox.test(indep  ~ dep)
  #  }
  #  if (sum(skewes_cond) == FALSE &  sd_cond == TRUE) {
  #    test = t.test(indep  ~ dep, var.equal = TRUE)
  #  }
  #  if (sum(skewes_cond) == FALSE &  sd_cond == FALSE) {
  #    test = t.test(indep  ~ dep)
  #  }
  #
  #}

  print("test")
  print(test)
  print("pval")
  print(rstatix::p_round(test$p.value, digits = 3))
```

```
}

es_num <- function(indep, dep, digits = 1) {
  #' Computes the hedges G
  #'
  #' The effect size is computed as M_{G2} - M_{G1} / s.
  #' (Hence why we need   #' to use the non-reverted outcome variable)
  #'
  #' @param indep The indepedent continuous variable
  #' @param dep The dependent variable (here == outcome)
  #' @param digits Nbr of digits to show prints/outputs (default = 2)
  print("Numeical Variable - Effect size")

  df_cd <- data.frame(d = indep, dep = dep)

  cd <- psych::cohen.d(df_cd, "dep")

  out <- round(cd$hedges.g, digits)

  #print(abs(out[2]))
  print(out)
}
```

```
report_cate <- function(indep, dep, indep_name, dep_name, digits=1) {
  #' Calcs descriptive stats for a categorical variable
  #'
  #'
  #' @param indep The indepedent continuous variable
  #' @param dep The dependent variable (here == outcome_reveresed)
  #' @param indep_name Name of the indep variable
  #' @param dep_name Name of the dep variable
  #' @param digits Nbr of digits to show prints/outputs (default = 2)

  print("Cate Variable - Reporting")


  tab_analysis <-
    table(indep,
          dep,
          dnn = c(indep_name, dep_name))

  ptab_analysis <- round(prop.table(tab_analysis, 2) * 100, digits)


  print(tab_analysis)
  print(ptab_analysis)

}

test_cate <- function(indep, dep, indep_name, dep_name, digits=1) {
  #' Run significant tests for a categorical variable
  #'
  #' @param indep The indepedent continuous variable
  #' @param dep The dependent variable (here == outcome_reveresed)
```

```r
  #' @param indep_name Name of the indep variable
  #' @param dep_name Name of the dep variable
  #' @param digits Nbr of digits to show prints/outputs (default = 2)

  print("Cate Variable - Testing")

  tab_analysis <-
    table(indep,
          dep,
          dnn = c(indep_name, dep_name))

  #chi_test_analysis <- chisq.test(tab_analysis, correct = FALSE)

  fisher_exact_test_analysis <- fisher.test(tab_analysis)

  print("Fisher's exact test")
  print(fisher_exact_test_analysis)
  print("pval")
  print(rstatix::p_round(fisher_exact_test_analysis$p.value, digits = 3))

}



es_binary <- function(indep, dep, indep_name, dep_name, digits=1) {
  #' Computes the Cohen's h + conf int
  #'
  #' The effect size is computed as:
  #' arc_sin_trans(prop_POB_group_1) - arc_sin_trans(prop_POB_group_2)
  #'
  #' @param indep The indepedent continuous variable
  #' @param dep The dependent variable (here == outcome)
  #' @param indep_name Name of the indep variable
  #' @param dep_name Name of the dep variable
  #' @param digits Nbr of digits to show prints/outputs (default = 2)

  print("Binary Variable - Effect size")

  tab_analysis <-
    table(indep,
          dep,
          dnn = c(indep_name, dep_name))

  #p1 = prop.table(tab_analysis, 1)[1,1]
  #p2 = prop.table(tab_analysis, 1)[2,1]
  #n1 = sum(tab_analysis[1,])
  #n2 = sum(tab_analysis[1,])

  #print(prop.table(tab_analysis, 1))

  p1 = prop.table(tab_analysis, 2)[1,1]
  p2 = prop.table(tab_analysis, 2)[1,2]
  n1 = sum(tab_analysis[,1])
  n2 = sum(tab_analysis[,2])
```

```r
  print(prop.table(tab_analysis, 2))


  cohens_h_val <- cohens_h(p1, p2, n1, n2)
  print(round(cohens_h_val, digits))
  print(round(cohens_h_val["h"], digits))



}

cohens_h = function( prop_1, prop_2, n1, n2, ci = 0.95 ){
  #' Computes the conf int for the cohans h stats
  #' Code from
  #' https://github.com/gasparl/neatstats/blob/master/R/props_neat.R#L458
  #' @param prop_1 Prop group 1
  #' @param prop_2 Prop group 2
  #' @param n1 N cases group 1
  #' @param n2 N cases group 2
  #' @param ci confidence level (default = 0.95)
  #' @return Vector with h, h_lower, h_upper

  x1 = asin(sign(prop_1) * sqrt(abs(prop_1)))

  x2 = asin(sign(prop_2) * sqrt(abs(prop_2)))

  es = x1 - x2

  se = sqrt(0.25 * (1 / n1 + 1 / n2 ))

  ci_diff = qnorm(1 - (1-ci) / 2) * se

  return( c( h = es*2, h_low = (es-ci_diff)*2, h_upp = (es+ci_diff)*2 ) )

}



es_cate <- function(indep, dep, indep_name, dep_name, digits=1) {
  #' Computes the Cramer's V
  #'
  #' @param indep The indepedent categorical variable
  #' @param dep The dependent variable (here == outcome)
  #' @param indep_name Name of the indep variable
  #' @param dep_name Name of the dep variable
  #' @param digits Nbr of digits to show prints/outputs (default = 2)
  print("Cate Variable - Effect size")

  tab_analysis <-
    table(indep,
          dep,
          dnn = c(indep_name, dep_name))

  print("cramersv")
  print(round(confintr::cramersv(tab_analysis), digits))
```

```
  print("ci_cramersv")
  print(confintr::ci_cramersv(tab_analysis), type = "bootstrap")

}
```

Load data

```
data <- readxl::read_excel("data/Liggare 221209 FINAL MEDSTAT 84 pat.xls")
```

Rm pat 7

```
data <- data[data$Patient != 7,]

stopifnot(length(unique(data$Patient)) == 83) # correct nbr unique pat
```

Fix levels and labels for categorical variables

```
data$Sex <-
  factor(data$Sex,
         levels = c(0, 1),
         labels = c("Male", "Female"))

data$`Hematological diagnosis` <-
  factor(
    data$`Hematological diagnosis`,
    levels = c(0, 1, 2, 3),
    labels = c("MDS/AML", "Myelom", "Lymfom", "other")
  )

data$CTCAE <-
  factor(
    data$CTCAE,
    levels = c(0, 1, 2),
    labels = c("CTCAE 2", "CTCAE 3", "CTCAE 4")
  )

data$`Platelet transfusion within 24hrs` <-
  factor(
    data$`Platelet transfusion within 24hrs`,
    levels = c(0, 1),
    labels = c("No", "Yes")
  )

data$`Type of dentoalveolar surgery` <-
  factor(
    data$`Type of dentoalveolar surgery`,
    levels = c(0, 1),
    labels = c("One", "gte 2")
  )

data$`Adjacent teeth` <-
  factor(data$`Adjacent teeth`,
         levels = c(0, 1),
```

```r
         labels = c("No", "Yes"))


# We leave data$`Extraction diagnosis` with numbers,
# since the labels ar very long. However, here we can
# check for labels that are easier to use...


data$`Extraction diagnosis` <- factor(data$`Extraction diagnosis`)

data$`Local hemostatic treatment` <-
  factor(
    data$`Local hemostatic treatment`,
    levels = c(0, 1, 2, 3),
    labels = c("No", "sutur", "local hemo", "sutur + local")
  )

data$POB <-
  factor(data$POB,
         levels = c(0, 1, 2),
         labels = c("No", "small", "larger"))

data$`first visit` <-
  factor(data$`first visit`,
         levels = c(0, 1),
         labels = c("Yes", "follow-up"))
```

Create the variable `visit` which shows the visit number for each patient

```r
data$visit <- 1

for (p in unique(data$Patient)) {
  data[data$Patient == p,]$visit <-
    1:length(data[data$Patient == p,]$`first visit`)
}
```

Create variable `outcome`, where we have `outcome = NPOB === POB = No` and `outcome = POB === POB = small/large` From https://stackoverflow.com/a/66295940.

```r
data <- data %>%
  dplyr::mutate(outcome = case_when(POB == "No" ~ "No",
                                    POB == "small" | POB == "larger" ~ "Yes"))

data$outcome <- factor(data$outcome)
```

```r
data <- data %>%
  dplyr::mutate(
    `Local hemostatic treatment yes/no` = case_when(
      `Local hemostatic treatment` == "No" ~ "No",
      `Local hemostatic treatment` == "sutur" |
        `Local hemostatic treatment` == "local hemo" |
        `Local hemostatic treatment` == "sutur + local" ~ "Yes"
    )
  )
```

```r
data$`Local hemostatic treatment yes/no` <-
  factor(data$`Local hemostatic treatment yes/no`)
```

Change order to get more interpretable results

```r
data$outcome_reversed <- relevel(data$outcome, "Yes")
data$`Platelet transfusion within 24hrs reversed` <-
  relevel(data$`Platelet transfusion within 24hrs`, "Yes")
data$`Local hemostatic treatment yes/no reversed` <-
  relevel(data$`Local hemostatic treatment yes/no`, "Yes")
data$`Adjacent teeth reversed` <-
  relevel(data$`Adjacent teeth`, "Yes")
data$`Sex reversed` <- relevel(data$Sex, "Female")
```

Analyses

Table 1/5 (Patient characteristics)

NB here we calc the patient characteristics for both first time and follow up visits.

Var: Sex

```r
report_cate(data$Sex,
            data$`first visit`,
            "Sex",
            "First visit")
```

```
[1] "Cate Variable - Reporting"
        First visit
Sex       Yes follow-up
  Male    57        18
  Female  26         5
        First visit
Sex         Yes follow-up
  Male   68.7      78.3
  Female 31.3      21.7
```

Var: Age

```r
report_num(data$Age, data$`first visit`)
```

```
[1] "Numeical Variable - Reporting"
[1] "median:"
dep: Yes
[1] 65
------------------------------------------------------------
dep: follow-up
[1] 67
[1] "range"
dep: Yes
[1] 24 85
------------------------------------------------------------
dep: follow-up
```

```
[1] 38 77
```

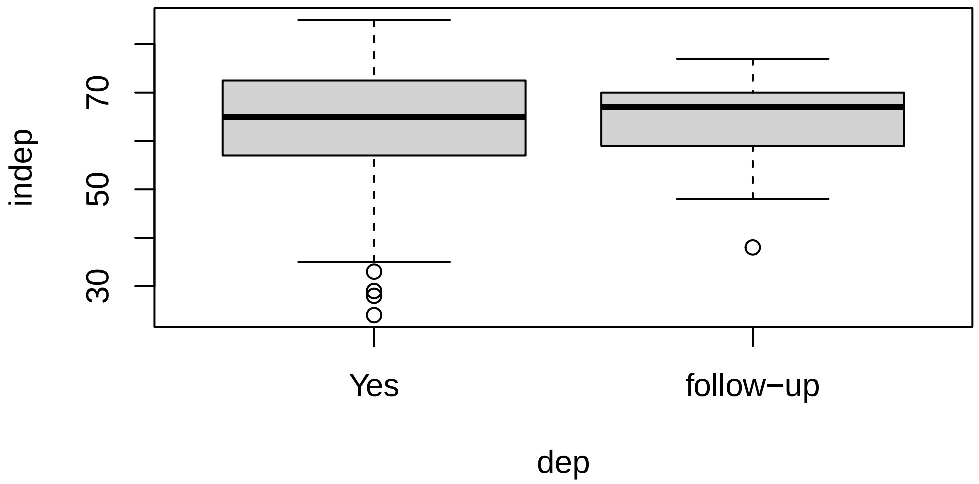

Var: Platelet count prior any transfusion

```
report_num(data$`Platelet count prior any transfusion`,
            data$`first visit`)
```

```
[1] "Numeical Variable - Reporting"
[1] "median:"
dep: Yes
[1] 34
------------------------------------------------------------
dep: follow-up
[1] 41
[1] "range"
dep: Yes
[1]  5 75
------------------------------------------------------------
dep: follow-up
[1]  5 72
```

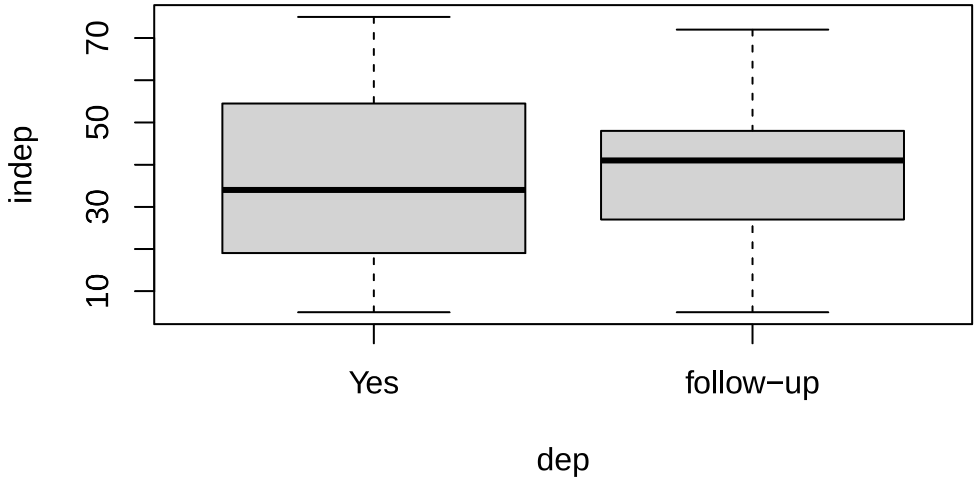

Var: Hematological diagnosis

```
report_cate(
  data$`Hematological diagnosis`,
  data$`first visit`,
  "Hematological diagnosis",
  "first visit"
)
```

```
[1] "Cate Variable - Reporting"
                       first visit
Hematological diagnosis Yes follow-up
                MDS/AML  65        14
                Myelom    4         5
                Lymfom    8         1
                other     6         3
                       first visit
Hematological diagnosis  Yes follow-up
                MDS/AML 78.3      60.9
                Myelom   4.8      21.7
                Lymfom   9.6       4.3
                other    7.2      13.0
```

Var: CTCAE

```
report_cate(data$CTCAE,
            data$`first visit`,
            "CTCAE",
            "first visit")
```

```
[1] "Cate Variable - Reporting"
         first visit
CTCAE     Yes follow-up
  CTCAE 2  22         4
```

```
  CTCAE 3  34        13
  CTCAE 4  27         6
         first visit
CTCAE      Yes follow-up
  CTCAE 2 26.5      17.4
  CTCAE 3 41.0      56.5
  CTCAE 4 32.5      26.1
```

Var: Platelet transfusion

```
report_cate(
  data$`Platelet transfusion within 24hrs reversed`,
  data$`first visit`,
  "Platelet transfusion",
  "fist visit"
)
```

```
[1] "Cate Variable - Reporting"
                    fist visit
Platelet transfusion Yes follow-up
                 Yes  48        11
                 No   35        12
                    fist visit
Platelet transfusion  Yes follow-up
                 Yes 57.8      47.8
                 No  42.2      52.2
```

Var: Local hemostatic treatment

```
report_cate(
  data$`Local hemostatic treatment yes/no reversed`,
  data$`first visit`,
  "Local hemostatic treatment yes/no",
  "first visit"
)
```

```
[1] "Cate Variable - Reporting"
                                 first visit
Local hemostatic treatment yes/no Yes follow-up
                              Yes  71        21
                              No   12         2
                                 first visit
Local hemostatic treatment yes/no  Yes follow-up
                              Yes 85.5      91.3
                              No  14.5       8.7
```

Var: Nbr teeth

```
report_num(data$`Number of teeth extracted`,
            data$`first visit`)
```

```
[1] "Numeical Variable - Reporting"
[1] "median:"
dep: Yes
[1] 1
------------------------------------------------------------
```

```
dep: follow-up
[1] 2
[1] "range"
dep: Yes
[1] 0 7
------------------------------------------------------------
dep: follow-up
[1] 1 5
```

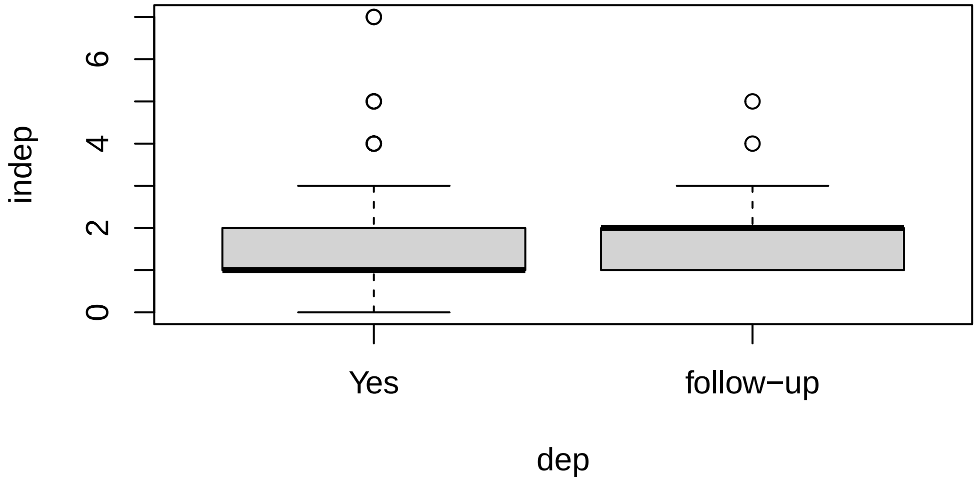

Var: Extraction diagnosis

```
report_cate(
  data$`Extraction diagnosis`,
  data$`first visit`,
  "Extraction diagnosis",
  "first visit"
)
```

```
[1] "Cate Variable - Reporting"
                    first visit
Extraction diagnosis Yes follow-up
                   0  31         8
                   1  33        13
                   2  16         2
                   3   1         0
                   4   2         0
                    first visit
Extraction diagnosis  Yes follow-up
                   0 37.3      34.8
                   1 39.8      56.5
                   2 19.3       8.7
                   3  1.2       0.0
                   4  2.4       0.0
```

Var: Type of dentalveolar surgery

```
report_cate(
  data$`Type of dentoalveolar surgery`,
  data$`first visit`,
  "Type of dentalveolar surgery",
  "first visit"
)
```

```
[1] "Cate Variable - Reporting"
                            first visit
Type of dentalveolar surgery Yes follow-up
                       One    55        11
                       gte 2  28        12
                            first visit
Type of dentalveolar surgery  Yes follow-up
                       One   66.3      47.8
                       gte 2 33.7      52.2
```

Var: Adjacent teeth

```
report_cate(
  data$`Adjacent teeth reversed`,
  data$`first visit`,
  "Adjacent teeth",
  "first visit"
)
```

```
[1] "Cate Variable - Reporting"
              first visit
Adjacent teeth Yes follow-up
           Yes  19         9
           No   64        14
              first visit
Adjacent teeth  Yes follow-up
           Yes 22.9      39.1
           No  77.1      60.9
```

Table 2 (Characteristics of base line and clinical variables with respect to the strata POB/NPOB)

Select pop for analyses

```
pop = "first_visit" # first_visit/CTCAE 2/ CTCAE 3/ CTCAE 4

if (pop == "first_visit"){
  data_pop <- data[data$`first visit` == "Yes", ]
}


if (pop == "CTCAE 2"){
  #data_pop <- data[data$`first visit` == "Yes", ]
  data_pop <- data[data$`first visit` == "Yes" & data$CTCAE == "CTCAE 2",]
}
```

```r
if (pop == "CTCAE 3"){
  #data_pop <- data[data$`first visit` == "Yes", ]
  data_pop <- data[data$`first visit` == "Yes" & data$CTCAE == "CTCAE 3",]
}


if (pop == "CTCAE 4"){
  #data_pop <- data[data$`first visit` == "Yes", ]
  data_pop <- data[data$`first visit` == "Yes" & data$CTCAE == "CTCAE 4",]
}
```

Get tot nbr of teeth

```r
sum(data_pop$`Number of teeth extracted`)
```

```
[1] 144
```

POB ~ Sex

```r
report_cate(data_pop$Sex,
          data_pop$outcome_reversed,
          "Sex",
          "POB (outcome)")
```

```
[1] "Cate Variable - Reporting"
        POB (outcome)
Sex       Yes No
  Male      5 52
  Female    0 26
        POB (outcome)
Sex         Yes    No
  Male    100.0  66.7
  Female    0.0  33.3
```

```r
test_cate(data_pop$Sex,
          data_pop$outcome_reversed,
          "Sex",
          "POB (outcome)")
```

```
[1] "Cate Variable - Testing"
[1] "Fisher's exact test"

    Fisher's Exact Test for Count Data

data:  tab_analysis
p-value = 0.3187
alternative hypothesis: true odds ratio is not equal to 1
95 percent confidence interval:
 0.4234372       Inf
sample estimates:
odds ratio
       Inf

[1] "pval"
[1] 0.319
```

```r
es_binary(data_pop$Sex,
          data_pop$outcome_reversed,
          "Sex",
          "POB (outcome)")
```

```
[1] "Binary Variable - Effect size"
        POB (outcome)
Sex              Yes        No
  Male   1.0000000 0.6666667
  Female 0.0000000 0.3333333
    h h_low h_upp
  1.2   0.3   2.1
  h
1.2
```

POB ~ Age

```r
by(data_pop$Age, data_pop$outcome_reversed, confintr::skewness)
```

```
data_pop$outcome_reversed: Yes
[1] -0.4895625
------------------------------------------------------------
data_pop$outcome_reversed: No
[1] -0.9048545
```

```r
report_num(data_pop$Age, data_pop$outcome_reversed)
```

```
[1] "Numeical Variable - Reporting"
[1] "median:"
dep: Yes
[1] 64
------------------------------------------------------------
dep: No
[1] 65.5
[1] "range"
dep: Yes
[1] 42 78
------------------------------------------------------------
dep: No
[1] 24 85
```

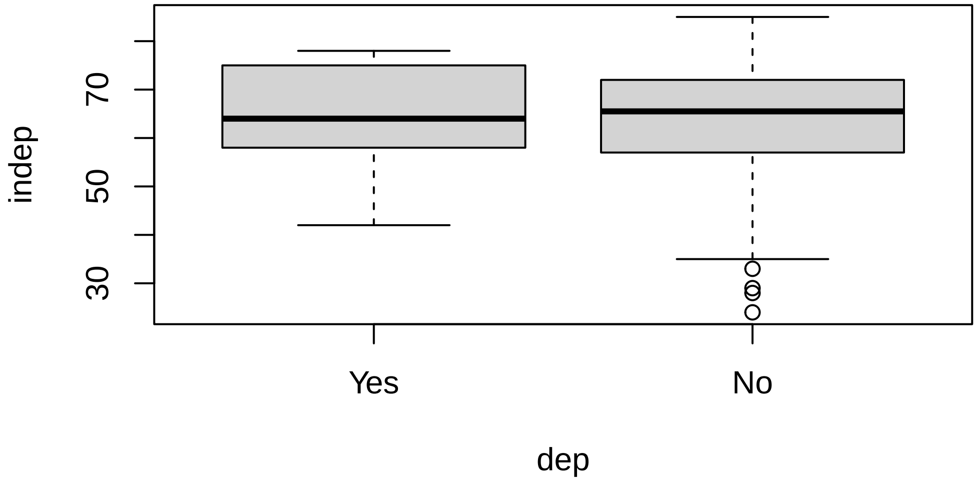


```r
test_num(data_pop$Age, data_pop$outcome_reversed)
```

```
[1] "Numeical Variable - Testing"
[1] FALSE
[1] FALSE
[1] TRUE
[1] "test"

    Wilcoxon rank sum test with continuity correction

data:  indep by dep
W = 201, p-value = 0.9161
alternative hypothesis: true location shift is not equal to 0

[1] "pval"
[1] 0.916
```

```r
es_num(data_pop$Age, data_pop$outcome)
```

```
[1] "Numeical Variable - Effect size"
  lower effect upper
d  -0.8    0.1     1
```

```r
skewes = by(data_pop$Age, data_pop$outcome_reversed, confintr::skewness)

 t.test(data_pop$Age  ~ data_pop$outcome_reversed, var.equal = TRUE)
```

```
    Two Sample t-test

data:  data_pop$Age by data_pop$outcome_reversed
t = 0.13568, df = 81, p-value = 0.8924
alternative hypothesis: true difference in means between group Yes and group No is not equal to 0
95 percent confidence interval:
 -11.77286  13.49594
```

```
sample estimates:
mean in group Yes  mean in group No 
         63.40000          62.53846 
```

POB ~ Platelet count prior any transfusion

```
report_num(data_pop$`Platelet count prior any transfusion`,
            data_pop$outcome_reversed)
```

```
[1] "Numeical Variable - Reporting"
[1] "median:"
dep: Yes
[1] 35
------------------------------------------------------------ 
dep: No
[1] 33.5
[1] "range"
dep: Yes
[1] 13 62
------------------------------------------------------------ 
dep: No
[1]  5 75
```

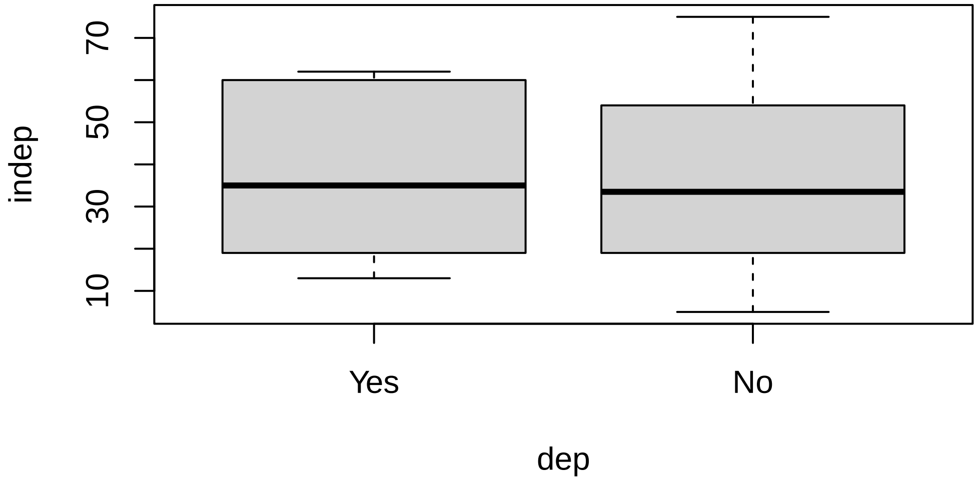


```
test_num(data_pop$`Platelet count prior any transfusion`,
          data_pop$outcome_reversed)
```

```
[1] "Numeical Variable - Testing"
[1] FALSE
[1] FALSE
[1] TRUE
[1] "test"

    Wilcoxon rank sum test with continuity correction
```

```
data:  indep by dep
W = 210, p-value = 0.7813
alternative hypothesis: true location shift is not equal to 0

[1] "pval"
[1] 0.781
```

```
es_num(data_pop$`Platelet count prior any transfusion`,
        data_pop$outcome)
```

```
[1] "Numeical Variable - Effect size"
  lower effect upper
d  -0.8    0.1     1
```

POB ~ Hematological diagnosis

```
report_cate(
  data_pop$`Hematological diagnosis`,
  data_pop$outcome_reversed,
  "Hematological diagnosis",
  "POB (outcome)"
)
```

```
[1] "Cate Variable - Reporting"
                       POB (outcome)
Hematological diagnosis Yes No
                MDS/AML   4 61
                Myelom    1  3
                Lymfom    0  8
                other     0  6
                       POB (outcome)
Hematological diagnosis  Yes   No
                MDS/AML 80.0 78.2
                Myelom  20.0  3.8
                Lymfom   0.0 10.3
                other    0.0  7.7
```

```
test_cate(
  data_pop$`Hematological diagnosis`,
  data_pop$outcome_reversed,
  "Hematological diagnosis",
  "POB (outcome)"
)
```

```
[1] "Cate Variable - Testing"
[1] "Fisher's exact test"

    Fisher's Exact Test for Count Data

data:  tab_analysis
p-value = 0.3891
alternative hypothesis: two.sided

[1] "pval"
[1] 0.389
```

```r
es_cate(
  data_pop$`Hematological diagnosis`,
  data_pop$outcome_reversed,
  "Hematological diagnosis",
  "POB (outcome)"
)
```

```
[1] "Cate Variable - Effect size"
[1] "cramersv"
```

```
Warning in stats::chisq.test(x, correct = correct): Chi-squared approximation
may be incorrect
```

```
[1] 0.2
[1] "ci_cramersv"
```

```
Warning in stats::chisq.test(x, correct = correct): Chi-squared approximation
may be incorrect
```

```
    Two-sided 95% chi-squared confidence interval for the population
    Cramer's V

Sample estimate: 0.2036889
Confidence interval:
    2.5%    97.5%
0.000000 0.419986
```

POB ~ CTCAE

```r
report_cate(data_pop$CTCAE,
            data_pop$outcome_reversed,
            "CTCAE",
            "POB (outcome)")
```

```
[1] "Cate Variable - Reporting"
         POB (outcome)
CTCAE      Yes No
  CTCAE 2    2 20
  CTCAE 3    1 33
  CTCAE 4    2 25
         POB (outcome)
CTCAE       Yes   No
  CTCAE 2 40.0 25.6
  CTCAE 3 20.0 42.3
  CTCAE 4 40.0 32.1
```

```r
test_cate(data_pop$CTCAE,
          data_pop$outcome_reversed,
          "CTCAE",
          "POB (outcome)")
```

```
[1] "Cate Variable - Testing"
[1] "Fisher's exact test"

    Fisher's Exact Test for Count Data

data:  tab_analysis
p-value = 0.6079
```

```
alternative hypothesis: two.sided

[1] "pval"
[1] 0.608
```

```
es_cate(data_pop$CTCAE,
        data_pop$outcome_reversed,
        "CTCAE",
        "POB (outcome)")
```

```
[1] "Cate Variable - Effect size"
[1] "cramersv"

Warning in stats::chisq.test(x, correct = correct): Chi-squared approximation
may be incorrect

[1] 0.1
[1] "ci_cramersv"

Warning in stats::chisq.test(x, correct = correct): Chi-squared approximation
may be incorrect

    Two-sided 95% chi-squared confidence interval for the population
    Cramer's V

Sample estimate: 0.1112675
Confidence interval:
     2.5%     97.5%
0.0000000 0.3300568
```

POB ~ Platelet transfusion

```
report_cate(
  data_pop$`Platelet transfusion within 24hrs reversed`,
  data_pop$outcome_reversed,
  "Platelet transfusion",
  "POB (outcome)"
)
```

```
[1] "Cate Variable - Reporting"
                    POB (outcome)
Platelet transfusion Yes No
                 Yes   2 46
                 No    3 32
                    POB (outcome)
Platelet transfusion Yes No
                 Yes  40 59
                 No   60 41
```

```
test_cate(
  data_pop$`Platelet transfusion within 24hrs reversed`,
  data_pop$outcome_reversed,
  "Platelet transfusion",
  "POB (outcome)"
)
```

```
[1] "Cate Variable - Testing"
[1] "Fisher's exact test"
```

```
    Fisher's Exact Test for Count Data

data:  tab_analysis
p-value = 0.6456
alternative hypothesis: true odds ratio is not equal to 1
95 percent confidence interval:
 0.03712301 4.32991873
sample estimates:
odds ratio
 0.4681642

[1] "pval"
[1] 0.646
```

```
es_binary(
  data_pop$`Platelet transfusion within 24hrs reversed`,
  data_pop$outcome_reversed,
  "Platelet transfusion",
  "POB (outcome)"
)
```

```
[1] "Binary Variable - Effect size"
                    POB (outcome)
Platelet transfusion       Yes        No
                 Yes 0.4000000 0.5897436
                 No  0.6000000 0.4102564
    h h_low h_upp
 -0.4  -1.3   0.5
   h
-0.4
```

POB ~ Local hemostatic treatment

```
report_cate(
  data_pop$`Local hemostatic treatment yes/no reversed`,
  data_pop$outcome_reversed,
  "Local hemostatic treatment yes/no",
  "POB (outcome)"
)
```

```
[1] "Cate Variable - Reporting"
                                 POB (outcome)
Local hemostatic treatment yes/no Yes No
                              Yes   5 66
                              No    0 12
                                 POB (outcome)
Local hemostatic treatment yes/no   Yes    No
                              Yes 100.0  84.6
                              No    0.0  15.4
```

```
test_cate(
  data_pop$`Local hemostatic treatment yes/no reversed`,
  data_pop$outcome_reversed,
  "Local hemostatic treatment yes/no",
```

```r
    "POB (outcome)"
  )
```

```
[1] "Cate Variable - Testing"
[1] "Fisher's exact test"

    Fisher's Exact Test for Count Data

data:  tab_analysis
p-value = 1
alternative hypothesis: true odds ratio is not equal to 1
95 percent confidence interval:
 0.1475694       Inf
sample estimates:
odds ratio
       Inf

[1] "pval"
[1] 1
```

```r
  es_binary(
    data_pop$`Local hemostatic treatment yes/no reversed`,
    data_pop$outcome_reversed,
    "Local hemostatic treatment yes/no",
    "POB (outcome)"
  )
```

```
[1] "Binary Variable - Effect size"
                                 POB (outcome)
Local hemostatic treatment yes/no       Yes        No
                              Yes 1.0000000 0.8461538
                              No  0.0000000 0.1538462
    h h_low h_upp
  0.8  -0.1   1.7
  h
0.8
```

POB ~ Nbr teeth

```r
  report_num(data_pop$`Number of teeth extracted`,
             data_pop$outcome_reversed)
```

```
[1] "Numeical Variable - Reporting"
[1] "median:"
dep: Yes
[1] 2
------------------------------------------------------------
dep: No
[1] 1
[1] "range"
dep: Yes
[1] 1 3
------------------------------------------------------------
dep: No
[1] 0 7
```

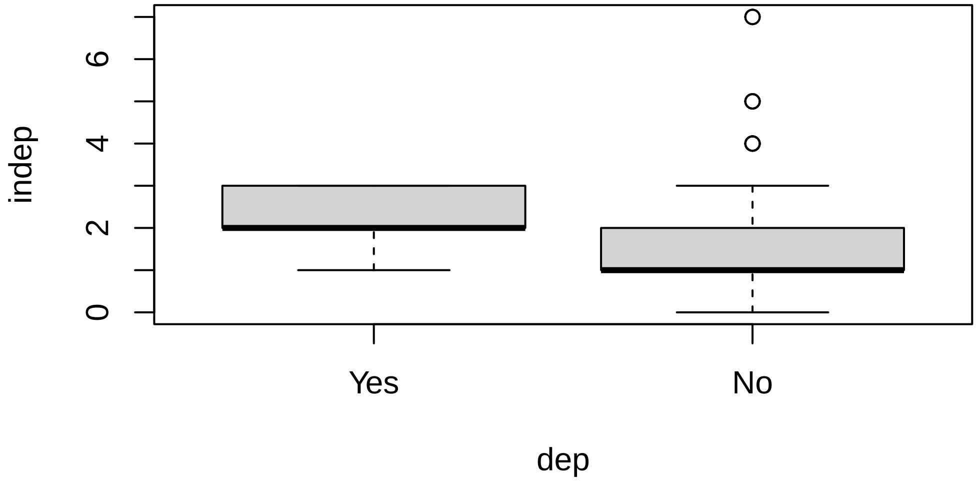


```
test_num(data_pop$`Number of teeth extracted`,
          data_pop$outcome_reversed)
```

```
[1] "Numeical Variable - Testing"
[1] TRUE
[1] FALSE
[1] TRUE
[1] "test"

    Wilcoxon rank sum test with continuity correction

data:  indep by dep
W = 277, p-value = 0.0691
alternative hypothesis: true location shift is not equal to 0

[1] "pval"
[1] 0.069
```

```
es_num(data_pop$`Number of teeth extracted`, data_pop$outcome)
```

```
[1] "Numeical Variable - Effect size"
  lower effect upper
d  -0.5    0.4   1.3
```

```
by(data_pop$`Number of teeth extracted`, data_pop$outcome_reversed, quantile)
```

```
data_pop$outcome_reversed: Yes
  0%  25%  50%  75% 100% 
   1    2    2    3    3 
------------------------------------------------------------
data_pop$outcome_reversed: No
  0%  25%  50%  75% 100% 
   0    1    1    2    7 
```

POB ~ Extraction diagnosis

```r
report_cate(
  data_pop$`Extraction diagnosis`,
  data_pop$outcome_reversed,
  "Extraction diagnosis",
  "POB (outcome)"
)
```

```
[1] "Cate Variable - Reporting"
                     POB (outcome)
Extraction diagnosis Yes No
                   0   1 30
                   1   3 30
                   2   0 16
                   3   1  0
                   4   0  2
                     POB (outcome)
Extraction diagnosis  Yes   No
                   0 20.0 38.5
                   1 60.0 38.5
                   2  0.0 20.5
                   3 20.0  0.0
                   4  0.0  2.6
```

```r
test_cate(
  data_pop$`Extraction diagnosis`,
  data_pop$outcome_reversed,
  "Extraction diagnosis",
  "POB (outcome)"
)
```

```
[1] "Cate Variable - Testing"
[1] "Fisher's exact test"

    Fisher's Exact Test for Count Data

data:  tab_analysis
p-value = 0.06204
alternative hypothesis: two.sided

[1] "pval"
[1] 0.062
```

```r
es_cate(
  data_pop$`Extraction diagnosis`,
  data_pop$outcome_reversed,
  "Extraction diagnosis",
  "POB (outcome)"
)
```

```
[1] "Cate Variable - Effect size"
[1] "cramersv"
```

```
Warning in stats::chisq.test(x, correct = correct): Chi-squared approximation
may be incorrect
```

```
[1] 0.5
[1] "ci_cramersv"
```

```
Warning in stats::chisq.test(x, correct = correct): Chi-squared approximation
may be incorrect
```

```
    Two-sided 95% chi-squared confidence interval for the population
    Cramer's V

Sample estimate: 0.4621959
Confidence interval:
     2.5%     97.5%
0.2882201 0.6804917
```

POB ~ Type of dentalveolar surgery

```
report_cate(
  data_pop$`Type of dentoalveolar surgery`,
  data_pop$outcome_reversed,
  "Type of dentalveolar surgery",
  "POB (outcome)"
)
```

```
[1] "Cate Variable - Reporting"
                             POB (outcome)
Type of dentalveolar surgery Yes No
                        One     1 54
                        gte 2   4 24
                             POB (outcome)
Type of dentalveolar surgery  Yes   No
                        One   20.0 69.2
                        gte 2 80.0 30.8
```

```
test_cate(
  data_pop$`Type of dentoalveolar surgery`,
  data_pop$outcome_reversed,
  "Type of dentalveolar surgery",
  "POB (outcome)"
)
```

```
[1] "Cate Variable - Testing"
[1] "Fisher's exact test"

    Fisher's Exact Test for Count Data

data:  tab_analysis
p-value = 0.04217
alternative hypothesis: true odds ratio is not equal to 1
95 percent confidence interval:
 0.002222231 1.233101957
sample estimates:
odds ratio
 0.1142747

[1] "pval"
[1] 0.042
```

```r
es_binary(
  data_pop$`Type of dentoalveolar surgery`,
  data_pop$outcome_reversed,
  "Type of dentalveolar surgery",
  "POB (outcome)"
)
```

```
[1] "Binary Variable - Effect size"
                            POB (outcome)
Type of dentalveolar surgery       Yes        No
                       One   0.2000000 0.6923077
                       gte 2 0.8000000 0.3076923
    h h_low h_upp
 -1.0  -1.9  -0.1
 h
-1
```

POB ~ Adjacent teeth

```r
report_cate(
  data_pop$`Adjacent teeth reversed`,
  data_pop$outcome_reversed,
  "Adjacent teeth",
  "POB (outcome)"
)
```

```
[1] "Cate Variable - Reporting"
              POB (outcome)
Adjacent teeth Yes No
           Yes   2 17
           No    3 61
              POB (outcome)
Adjacent teeth  Yes   No
           Yes 40.0 21.8
           No  60.0 78.2
```

```r
test_cate(
  data_pop$`Adjacent teeth reversed`,
  data_pop$outcome_reversed,
  "Adjacent teeth",
  "POB (outcome)"
)
```

```
[1] "Cate Variable - Testing"
[1] "Fisher's exact test"

    Fisher's Exact Test for Count Data

data:  tab_analysis
p-value = 0.3216
alternative hypothesis: true odds ratio is not equal to 1
95 percent confidence interval:
  0.1835135 22.4158624
sample estimates:
odds ratio
  2.362268
```

```
[1] "pval"
[1] 0.322
```

```
es_binary(
  data_pop$`Adjacent teeth reversed`,
  data_pop$outcome_reversed,
  "Adjacent teeth",
  "POB (outcome)"
)
```

```
[1] "Binary Variable - Effect size"
              POB (outcome)
Adjacent teeth       Yes        No
           Yes 0.4000000 0.2179487
           No  0.6000000 0.7820513
    h h_low h_upp
  0.4  -0.5   1.3
  h
0.4
```

Table 3 (Characteristics of the variables post operative bleeding and PLT count before any transfusion with respect to the strata transfusion/no transfusion)

Risk of POB transfusion/no transfusion

```
data_pop$`Platelet transfusion within 24hrs (reverse)` <-
  relevel(data_pop$`Platelet transfusion within 24hrs`, "Yes")
```

```
report_cate(
  data_pop$outcome_reversed,
  data_pop$`Platelet transfusion within 24hrs (reverse)`,
  "POB (outcome)",
  "Platelet transfusion"
)
```

```
[1] "Cate Variable - Reporting"
             Platelet transfusion
POB (outcome) Yes No
          Yes   2  3
          No   46 32
             Platelet transfusion
POB (outcome)  Yes   No
          Yes  4.2  8.6
          No  95.8 91.4
```

```
test_cate(
  data_pop$outcome_reversed,
  data_pop$`Platelet transfusion within 24hrs (reverse)`,
  "POB (outcome)",
  "Platelet transfusion"
)
```

```
[1] "Cate Variable - Testing"
```

```
[1] "Fisher's exact test"

	Fisher's Exact Test for Count Data

data:  tab_analysis
p-value = 0.6456
alternative hypothesis: true odds ratio is not equal to 1
95 percent confidence interval:
 0.03712301 4.32991873
sample estimates:
odds ratio
 0.4681642

[1] "pval"
[1] 0.646
```

```
es_binary(
  data_pop$outcome_reversed,
  data_pop$`Platelet transfusion within 24hrs (reverse)`,
  "POB (outcome)",
  "Platelet transfusion"
)
```

```
[1] "Binary Variable - Effect size"
             Platelet transfusion
POB (outcome)        Yes         No
          Yes 0.04166667 0.08571429
          No  0.95833333 0.91428571
    h h_low h_upp
 -0.2  -0.6   0.3
   h
-0.2
```

Platelet count prior any transfusion

```
report_num(
  data_pop$`Platelet count prior any transfusion`,
  data_pop$`Platelet transfusion within 24hrs reversed`
)
```

```
[1] "Numeical Variable - Reporting"
[1] "median:"
dep: Yes
[1] 26.5
------------------------------------------------------------
dep: No
[1] 48
[1] "range"
dep: Yes
[1]  5 72
------------------------------------------------------------
dep: No
[1]  9 75
```

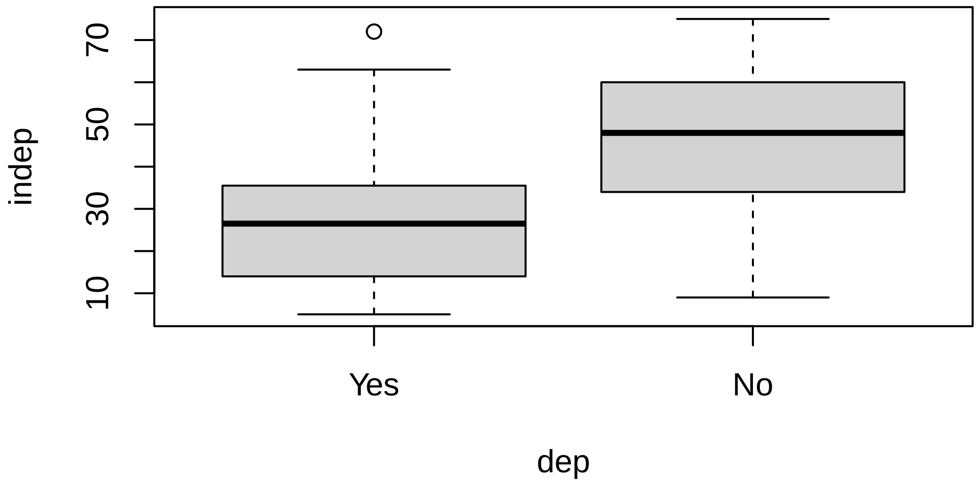


```
test_num(
  data_pop$`Platelet count prior any transfusion`,
  data_pop$`Platelet transfusion within 24hrs reversed`
)
```

```
[1] "Numeical Variable - Testing"
[1] FALSE
[1] FALSE
[1] TRUE
```

```
Warning in wilcox.test.default(x = DATA[[1L]], y = DATA[[2L]], ...): cannot
compute exact p-value with ties
```

```
[1] "test"

    Wilcoxon rank sum test with continuity correction

data:  indep by dep
W = 373.5, p-value = 1.72e-05
alternative hypothesis: true location shift is not equal to 0

[1] "pval"
[1] 1.72e-05
```

```
es_num(
  data_pop$`Platelet count prior any transfusion`,
  data_pop$`Platelet transfusion within 24hrs`
)
```

```
[1] "Numeical Variable - Effect size"
  lower effect upper
d  -1.6   -1.1  -0.6
```

Fig 2 (paths follow-up visits)

```
data_model_follow_up <- data[data$`first visit` == "follow-up", ]

patient_ids = unique(data_model_follow_up$Patient)

data$CTCAE_plotting <-
  as.numeric(factor(data$CTCAE,
                    levels = c("CTCAE 2", "CTCAE 3", "CTCAE 4"),
                    labels = c(2,3,4))) + 1
```

```
#windows()

png(file=paste(getwd(), "/results/figs/fig_2_240612.png", sep = ""),
    width=1000,
    height=1200)


par(mfrow = c(5, 3), mar = c(5, 5, 5, 5),cex.axis=1.0)

cex.axis <- 1.8

idx = 1
for (row in 1:5) {
  for (col in 1:3) {
    temp_x <- data[data$Patient == patient_ids[idx],]$visit
    temp_y <- data[data$Patient == patient_ids[idx],]$outcome
    temp_type_surg <-
      as.numeric(data[data$Patient ==
                  patient_ids[idx],]$`Type of dentoalveolar surgery`)
    temp_ctcae <- data[data$Patient == patient_ids[idx],]$CTCAE_plotting
    temp_trans <-
      data[data$Patient ==
             patient_ids[idx],]$`Platelet transfusion within 24hrs`

    print("---")
    print(temp_x)
    print(temp_type_surg)
    print(temp_ctcae)

    plot(
      temp_x,
      temp_type_surg,
      pch = 16,
      ylab = "",
      xlab = "",
      ylim = c(-0.3, 5),
      xlim = c(min(temp_x) - 0.1, max(temp_x) + 0.1),
      type = "l",
      col = "black",
      yaxt = 'n',
      xaxt = 'n',
      #main = paste("Pt. id: ", as.character(patient_ids[idx]))
    )
```

```r
mtext(paste("Pt. id: ", as.character(patient_ids[idx])),
      side = 3, line = 2.5, cex=1.1)

axis(1,
     at = temp_x,
     labels = temp_x,
     col = "black",
     cex.axis=cex.axis)

axis(
  2,
  at = c(1,2),
  labels = c("1", "  2"),
  col = "black",
  las = 3,
  cex.axis=cex.axis
)

if (col == 1) {
  mtext("Type of Dentoalveolar Surgery", side = 2, line = 2.5, cex=1.2)
} else {
  ylab = ""
}

if (col == 3) {
  mtext("CTCAE", side = 4, line = 2.5, cex=1.2)
} else {
  ylab = ""
}


if (row == 5) {
  mtext("Visit", side = 1, line = 2.5, cex=1.2)
} else {
  xlab = ""
}


lines(
  temp_x,
  temp_ctcae,
  pch = 16,
  ylab = "",
  xlab = "",
  type = "l",
  col = "black",
  yaxt = 'n',
  lty = 2 # dashed line
)

axis(
  4,
  at = c(2, 3, 4),
  labels = c(2, 3, 4),
```

```
        col = "black",
        col.axis = "black",
        las = 1,
        cex.axis=1.5
      )


      for (pos in 1:length(temp_x)) {
        if (temp_y[pos] == "Yes") {
          pob = " + POB"
        } else {
          pob = ""
        }
        if (temp_trans[pos] == "Yes") {
          text(temp_x[pos],
               -0.2,
               labels = paste("T", pob, sep = ""),
               cex = 1.5)
        } else {
          text(temp_x[pos],
               -0.2,
               labels = paste("~T", pob, sep = ""),
               cex = 1.5)
        }
      }


      idx = idx + 1

    }
  }
```

```
[1] "---"
[1] 1 2 3 4
[1] 2 2 1 2
[1] 3 3 3 3

[1] "---"
[1] 1 2 3
[1] 2 2 2
[1] 2 3 3

[1] "---"
[1] 1 2
[1] 1 1
[1] 2 2

[1] "---"
[1] 1 2 3
[1] 1 1 1
[1] 3 3 3

[1] "---"
[1] 1 2
[1] 2 2
[1] 3 3

[1] "---"
[1] 1 2
```

```
[1] 1 1
[1] 2 2

[1] "---"
[1] 1 2 3 4 5
[1] 2 2 2 2 2
[1] 3 3 2 4 3

[1] "---"
[1] 1 2
[1] 1 1
[1] 4 3

[1] "---"
[1] 1 2
[1] 1 1
[1] 2 4

[1] "---"
[1] 1 2
[1] 1 2
[1] 4 4

[1] "---"
[1] 1 2
[1] 2 1
[1] 4 4

[1] "---"
[1] 1 2
[1] 2 2
[1] 2 3

[1] "---"
[1] 1 2 3
[1] 1 1 1
[1] 4 4 4

[1] "---"
[1] 1 2
[1] 2 2
[1] 2 3

[1] "---"
[1] 1 2
[1] 2 1
[1] 2 2
```

```
#mtext("Outcomes for Follow-up Visits",
#      side = 3,
#      line = -1,
#      outer = TRUE,
#      cex=1.2)

dev.off()
```

```
pdf
  2
```

Table 4 (Model w all obs)

Mixed GLM with Temporal Correlation

```
data$Platelet_transfusion_within_24hrs_reversed <-
  data$`Platelet transfusion within 24hrs reversed`

data$Platelet_transfusion_within_24hrs <-
  data$`Platelet transfusion within 24hrs`

data$Type_of_dentoalveolar_surgery <- data$`Type of dentoalveolar surgery`

data$Patient_factor <- factor(data$Patient)

data$outcome_num <- as.numeric(data$outcome) - 1
```

Test with strucGlmer and glmmTMB + REML. Convergence problems for some models. **NB not included**

```
f <- as.formula(paste("outcome_num ~ ",
                      "CTCAE + ",
                      "(1 |Patient) +",
                      "nlmeCorStruct(1, corObj = corObj, sig = 1)"))
corObj <- nlme::Initialize(nlme::corAR1(0, form = ~ 1 | Patient), data)
fit_1_lme4ord <- strucGlmer(f, family = binomial, data = data)

f <- as.formula(paste("outcome_num ~ ",
                      "Platelet_transfusion_within_24hrs + ",
                      "(1 |Patient) +",
                      "nlmeCorStruct(1, corObj = corObj, sig = 1)"))
fit_2_lme4ord <- strucGlmer(f, family = binomial, data = data)

f <- as.formula(paste("outcome_num ~ ",
                      "Type_of_dentoalveolar_surgery + ",
                      "(1 |Patient) +",
                      "nlmeCorStruct(1, corObj = corObj, sig = 1)"))
fit_3_lme4ord <- strucGlmer(f, family = binomial, data = data)

f <- as.formula(paste("outcome_num ~ ",
                      "CTCAE + ",
                      "(1 |Patient) +",
                      "ar1(factor(visit) + 0 |Patient)"))
fit_1 <- glmmTMB(f, data = data, family = binomial, REML = TRUE)

f <- as.formula(paste("outcome_num ~ ",
                      "Platelet_transfusion_within_24hrs + ",
                      "(1 |Patient) +",
                      "ar1(factor(visit) + 0 |Patient)"))
fit_2 <- glmmTMB(f, data = data, family = binomial, REML = TRUE)
```

```
Warning in finalizeTMB(TMBStruc, obj, fit, h, data.tmb.old): Model convergence
problem; singular convergence (7). See vignette('troubleshooting'),
help('diagnose')
```

```r
f <- as.formula(paste("outcome_num ~ ",
                      "Type_of_dentoalveolar_surgery + ",
                      "(1 |Patient) +",
                      "ar1(factor(visit) + 0 |Patient)"))
fit_3 <- glmmTMB(f, data = data, family = binomial, REML = TRUE)
```

Run Penalized Quasi-Likelihood (this method is preferred since it seems to be the most robust method). It also overall seems to agree with the other methods, but without convergence issues.

```r
fit1 <- MASS::glmmPQL(
  outcome_num ~ CTCAE,
  random =  ~ 1  | Patient,
  data = data,
  family = binomial,
  correlation = nlme::corAR1(0.2, form = ~ 1 | Patient),
  verbose = TRUE
)
```

```
iteration 1
```

```
iteration 2
```

```r
fit2 <- MASS::glmmPQL(
  outcome_num ~ Platelet_transfusion_within_24hrs,
  random =  ~ 1 | Patient,
  data = data,
  family = binomial,
  correlation = nlme::corAR1(0.2, form = ~ 1 | Patient),
  verbose = TRUE
)
```

```
iteration 1
iteration 2
```

```r
fit3 <- MASS::glmmPQL(
  outcome_num ~ Type_of_dentoalveolar_surgery,
  random =  ~ 1 | Patient,
  data = data,
  family = binomial,
  correlation = nlme::corAR1(0.2, form = ~ 1 | Patient),
  verbose = TRUE
)
```

```
iteration 1
iteration 2
```

```r
digits_print = 2
options(digits=digits_print)
summary(fit1)
```

```
Linear mixed-effects model fit by maximum likelihood
  Data: data
  AIC BIC logLik
   NA  NA     NA

Random effects:
```

```
 Formula: ~1 | Patient
        (Intercept) Residual
StdDev:     0.00053     0.99

Correlation Structure: AR(1)
 Formula: ~1 | Patient
 Parameter estimate(s):
   Phi
-0.064
Variance function:
 Structure: fixed weights
 Formula: ~invwt
Fixed effects:  outcome_num ~ CTCAE
             Value Std.Error DF t-value p-value
(Intercept)  -2.48      0.73 82    -3.4  0.0011
CTCAECTCAE 3 -0.57      1.01 21    -0.6  0.5778
CTCAECTCAE 4  0.18      0.95 21     0.2  0.8477
 Correlation:
             (Intr) CTCAE3
CTCAECTCAE 3 -0.73
CTCAECTCAE 4 -0.77   0.56

Standardized Within-Group Residuals:
  Min    Q1   Med    Q3   Max
-0.32 -0.32 -0.29 -0.22  4.67

Number of Observations: 106
Number of Groups: 83
```

```
round(coef(summary(fit1))[,'Value'], 1)
```

```
 (Intercept) CTCAECTCAE 3 CTCAECTCAE 4
        -2.5         -0.6          0.2
```

```
round(coef(summary(fit1))[,'Std.Error'], 1)
```

```
 (Intercept) CTCAECTCAE 3 CTCAECTCAE 4
         0.7          1.0          0.9
```

```
round(coef(summary(fit1))[,'p-value'],3)
```

```
 (Intercept) CTCAECTCAE 3 CTCAECTCAE 4
       0.001        0.578        0.848
```

```
summary(fit2)
```

```
Linear mixed-effects model fit by maximum likelihood
  Data: data
  AIC BIC logLik
   NA  NA     NA

Random effects:
 Formula: ~1 | Patient
        (Intercept) Residual
StdDev:     0.00037     0.99
```

```
Correlation Structure: AR(1)
 Formula: ~1 | Patient
 Parameter estimate(s):
  Phi
-0.07
Variance function:
 Structure: fixed weights
 Formula: ~invwt
Fixed effects:  outcome_num ~ Platelet_transfusion_within_24hrs
                                     Value Std.Error DF t-value p-value
(Intercept)                          -2.38      0.51 82    -4.6    0.00
Platelet_transfusion_within_24hrsYes -0.51      0.77 22    -0.7    0.52
 Correlation:
                                     (Intr)
Platelet_transfusion_within_24hrsYes -0.67

Standardized Within-Group Residuals:
  Min    Q1   Med    Q3   Max
-0.31 -0.31 -0.24 -0.24  4.28

Number of Observations: 106
Number of Groups: 83
```

```
round(coef(summary(fit2))[,'Value'], 1)
```

```
                         (Intercept) Platelet_transfusion_within_24hrsYes
                                -2.4                                 -0.5
```

```
round(coef(summary(fit2))[,'Std.Error'], 1)
```

```
                         (Intercept) Platelet_transfusion_within_24hrsYes
                                 0.5                                  0.8
```

```
round(coef(summary(fit2))[,'p-value'],3)
```

```
                         (Intercept) Platelet_transfusion_within_24hrsYes
                                0.00                                 0.52
```

```
summary(fit3)
```

```
Linear mixed-effects model fit by maximum likelihood
  Data: data
  AIC BIC logLik
   NA  NA     NA

Random effects:
 Formula: ~1 | Patient
        (Intercept) Residual
StdDev:       3e-04     0.99

Correlation Structure: AR(1)
 Formula: ~1 | Patient
 Parameter estimate(s):
  Phi
-0.21
Variance function:
```

```
 Structure: fixed weights
 Formula: ~invwt
Fixed effects:  outcome_num ~ Type_of_dentoalveolar_surgery
                                   Value Std.Error DF t-value p-value
(Intercept)                         -4.1      0.97 82    -4.3   0.000
Type_of_dentoalveolar_surgerygte 2   2.4      1.06 22     2.3   0.033
 Correlation:
                                   (Intr)
Type_of_dentoalveolar_surgerygte 2 -0.92

Standardized Within-Group Residuals:
  Min    Q1   Med    Q3   Max
-0.42 -0.42 -0.13 -0.13  8.00

Number of Observations: 106
Number of Groups: 83
```

```
round(coef(summary(fit3))[,'Value'], 1)
```

```
                       (Intercept) Type_of_dentoalveolar_surgerygte 2
                              -4.1                                2.4
```

```
round(coef(summary(fit3))[,'Std.Error'], 1)
```

```
                       (Intercept) Type_of_dentoalveolar_surgerygte 2
                               1.0                                1.1
```

```
round(coef(summary(fit3))[,'p-value'],3)
```

```
                       (Intercept) Type_of_dentoalveolar_surgerygte 2
                             0.000                              0.033
```

Check auc

```
d = data.frame(cbind(data$outcome_num, predict(fit1, data, type="response")))
r1 <- pROC::roc(d$X1,d$X2,quiet = FALSE)
```

```
Setting levels: control = 0, case = 1
```

```
Setting direction: controls < cases
```

```
pROC::plot.roc(r1)
```

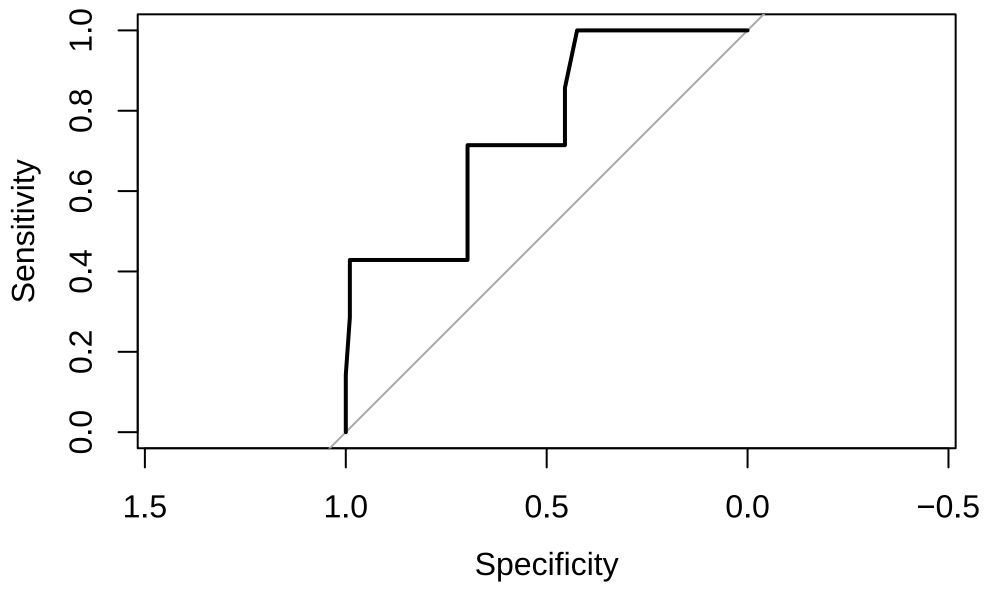


```
d = data.frame(cbind(data$outcome_num, predict(fit2, data, type="response")))
r2 <- pROC::roc(d$X1,d$X2,quiet = FALSE)
```

```
Setting levels: control = 0, case = 1
Setting direction: controls < cases
```

```
pROC::plot.roc(r2)
```

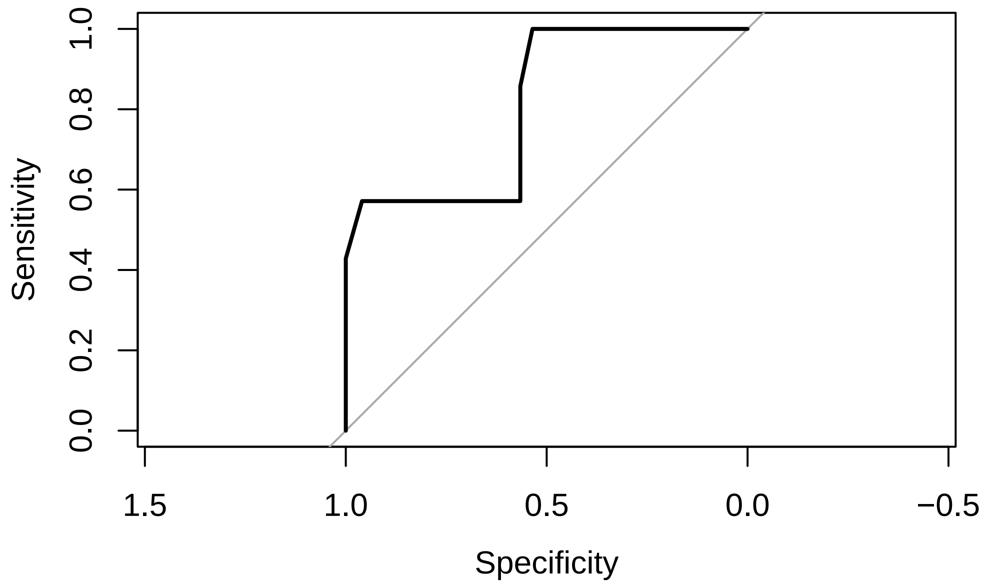


```
d = data.frame(cbind(data$outcome_num, predict(fit3, data, type="response")))
r3 <- pROC::roc(d$X1,d$X2,quiet = FALSE)
```

```
Setting levels: control = 0, case = 1
Setting direction: controls < cases
```

```
pROC::plot.roc(r3)
```

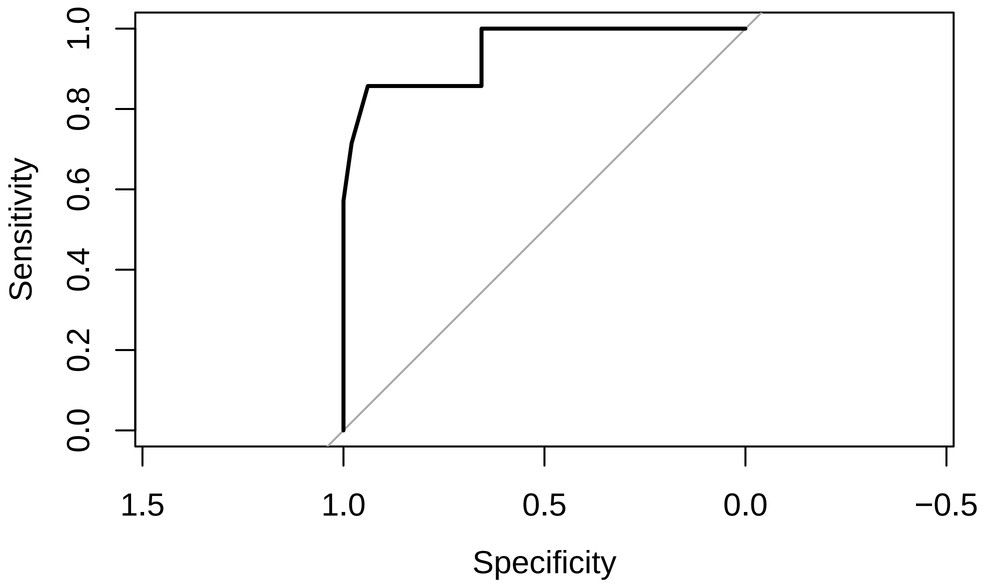


```
c(auc(r1), auc(r2), auc(r3))
```

```
[1] 0.75 0.81 0.94
```

```
rbind(ci.auc(r1), ci.auc(r2), ci.auc(r3))
```

```
     [,1] [,2] [,3]
[1,] 0.56 0.75 0.94
[2,] 0.63 0.81 0.99
[3,] 0.85 0.94 1.00
```

Check Nakagawa's R2

```
performance::r2_nakagawa(fit1)
```

```
# R2 for Mixed Models

  Conditional R2: 0.035
     Marginal R2: 0.035
```

```
performance::r2_nakagawa(fit2)
```

```
# R2 for Mixed Models

  Conditional R2: 0.019
     Marginal R2: 0.019
```

```
performance::r2_nakagawa(fit3)
```

```
# R2 for Mixed Models

  Conditional R2: 0.296
     Marginal R2: 0.296
```

(Naive) Robust GLM (NB not included)

```r
data$outcome_num = as.numeric(data$outcome) - 1

fit1_glm = glm(outcome_num ~ CTCAE,
               data = data,
               family = binomial) #(link = "probit"))

coef1 = lmtest::coeftest(fit1_glm,
                         vcov. = sandwich::vcovHC(fit1_glm, type = "HC0"))

fit2_glm = glm(outcome ~ `Platelet transfusion within 24hrs` ,
               data = data,
               family = binomial) #(link = "probit"))

coef2 = lmtest::coeftest(fit2_glm,
                         vcov. = sandwich::vcovHC(fit2_glm, type = "HC0"))


fit3_glm = glm(outcome ~ `Type of dentoalveolar surgery`,
               data = data,
               family = binomial) #(link = "probit"))

coef3 = lmtest::coeftest(fit3_glm,
                         vcov. = sandwich::vcovHC(fit3_glm, type = "HC0"))


# print est coefs
print(round(coef1, 2))
```

```
z test of coefficients:

             Estimate Std. Error z value Pr(>|z|)
(Intercept)     -2.48       0.74   -3.38   <2e-16 ***
CTCAECTCAE 3    -0.63       1.03   -0.61     0.54
CTCAECTCAE 4     0.18       0.95    0.19     0.85
---
Signif. codes:  0 '***' 0.001 '**' 0.01 '*' 0.05 '.' 0.1 ' ' 1
```

```r
print(round(coef2, 2))
```

```
z test of coefficients:

                                       Estimate Std. Error z value Pr(>|z|)
(Intercept)                               -2.37       0.52   -4.54   <2e-16 ***
`Platelet transfusion within 24hrs`Yes    -0.55       0.79   -0.70     0.48
---
Signif. codes:  0 '***' 0.001 '**' 0.01 '*' 0.05 '.' 0.1 ' ' 1
```

```r
print(round(coef3, 2))
```

```
z test of coefficients:

                                     Estimate Std. Error z value Pr(>|z|)
(Intercept)                             -4.17       1.01   -4.14   <2e-16 ***
`Type of dentoalveolar surgery`gte 2     2.44       1.10    2.22     0.03 *
---
Signif. codes:  0 '***' 0.001 '**' 0.01 '*' 0.05 '.' 0.1 ' ' 1
```

Compare models

```r
print(fit_3_lme4ord)
```

```
Structured GLMM fit by maximum likelihood (Laplace Approx)   ['strucGlmer']
 Family: binomial  ( logit )
Formula: f
   Data: data
     AIC      BIC   logLik deviance df.resid
    43.4     56.7    -16.7      6.1      101
Random effects term (class: unstruc):
  covariance parameter:   1.2
  variance-correlation:
 Groups  Name        Std.Dev.
 Patient (Intercept) 1.22
Random effects term (class: nlmeCorStruct):
Correlation structure of class corAR1 representing
Phi
 -1
  Standard deviation multiplier:  -15
Fixed Effects:
                       (Intercept)  Type_of_dentoalveolar_surgerygte 2
                            -10.30                                1.52
```

```r
summary(fit_3)
```

```
 Family: binomial  ( logit )
Formula:
outcome_num ~ Type_of_dentoalveolar_surgery + (1 | Patient) +
    ar1(factor(visit) + 0 | Patient)
Data: data

     AIC      BIC   logLik deviance df.resid
      52       65      -21       42      103

Random effects:

Conditional model:
 Groups    Name           Variance Std.Dev. Corr
 Patient   (Intercept)    4.31e-09 6.57e-05
 Patient.1 factor(visit)1 3.86e-01 6.22e-01 -0.78 (ar1)
Number of obs: 106, groups:  Patient, 83

Conditional model:
                                   Estimate Std. Error z value Pr(>|z|)
(Intercept)                           -4.17       1.01   -4.13  3.6e-05 ***
Type_of_dentoalveolar_surgerygte 2     2.43       1.11    2.18    0.029 *
---
Signif. codes:  0 '***' 0.001 '**' 0.01 '*' 0.05 '.' 0.1 ' ' 1
```

```r
summary(fit3)
```

```
Linear mixed-effects model fit by maximum likelihood
  Data: data
  AIC BIC logLik
   NA  NA     NA

Random effects:
 Formula: ~1 | Patient
        (Intercept) Residual
StdDev:       3e-04     0.99

Correlation Structure: AR(1)
 Formula: ~1 | Patient
 Parameter estimate(s):
  Phi
-0.21
Variance function:
 Structure: fixed weights
 Formula: ~invwt
Fixed effects:  outcome_num ~ Type_of_dentoalveolar_surgery
                                   Value Std.Error DF t-value p-value
(Intercept)                         -4.1      0.97 82    -4.3   0.000
Type_of_dentoalveolar_surgerygte 2   2.4      1.06 22     2.3   0.033
 Correlation:
                                   (Intr)
Type_of_dentoalveolar_surgerygte 2 -0.92

Standardized Within-Group Residuals:
  Min    Q1   Med    Q3   Max
-0.42 -0.42 -0.13 -0.13  8.00

Number of Observations: 106
Number of Groups: 83
```

```r
print(coef3)
```

```
z test of coefficients:

                                     Estimate Std. Error z value Pr(>|z|)
(Intercept)                             -4.17       1.01   -4.14  3.4e-05 ***
`Type of dentoalveolar surgery`gte 2     2.44       1.10    2.22    0.027 *
---
Signif. codes:  0 '***' 0.001 '**' 0.01 '*' 0.05 '.' 0.1 ' ' 1
```

How to Generated pdf

Steps for creating pdf from qmd file:

- Render
- Run `require("tinytex")`
- Replace `\author{Samuel Wiqvist}` with `\author{Samuel Wiqvist\footnote{Department of Learning, Informatics, Management \& Ethics (LIME), Karolinska Institutet, Stockholm, Sweden. Mail: samuel.wiqvist@ki.se}}` in the `.tex` file

- Run `xelatex('<file name>.tex')xelatex('<file name>.tex')`
