# Supplementary material for: Postoperative bleeding after dentoalveolar surgery in patients with thrombocytopenia—are prophylactic platelet transfusions necessary?
Source: Support Care Cancer. 2024 Oct 7;32(10):703. doi: 10.1007/s00520-024-08917-1 (PMC11456549; doi:10.1007/s00520-024-08917-1)
Supplement: Supplementary file 2 — Supplementary file2 (DOCX 2331 KB) [file 520_2024_8917_MOESM2_ESM.docx]

**Postoperative bleeding after dentoalveolar surgery in patients with thrombocytopenia - are prophylactic platelet transfusions necessary?**

Johan Lundström^1, 2^, Samuel Wiqvist^3^, Martin Jädersten^4, 5^, Victor Tollemar^1^, Karin Garming Legert^1^

**Affiliations**

^1^Department of Dental Medicine, Karolinska Institutet, Stockholm, Sweden
^2^Public Dental Health Service Stockholm AB, Stockholm, Sweden
^3^Department of Learning, Informatics, Management & Ethics (LIME), Karolinska Institutet, Stockholm, Sweden
^4^Department of Hematology, Karolinska University Hospital, Stockholm, Sweden
^5^Center for Hematology and Regenerative Medicine (HERM), Karolinska Institutet, Stockholm, Sweden

Johan Lundström: johan.n.lundstrom@regionstockholm.se

**Supplementary information Patients characteristics additional visits**

**Table 5** Patient characteristics for the additional visits (*n* = 15 patients, 23 additional visits). Categorical variables are reported as counts (percentages), and numerical variables are reported as medians (ranges). All results are reported to a precision of one digit.

| Variable |  |
| --- | --- |
| Gender, n (%) |  |
| Male | 18 (78.3) |
| Female | 5 (21.7) |
| Age (years), median (range) | 67 (38, 77) |
| PLT count prior to any transfusion, (*10^9^/L), median (range) | 41 (5, 72) |
| Hematological diagnosis n, (%) |  |
| MDS/AML | 14 (60.9) |
| Myeloma | 5 (21.7) |
| Lymphoma^a^ | 1 (4.3) |
| Other^b^ | 3 (13) |
| CTCAE, thrombocytopenia severity (*10^9^/L), n (%) |  |
| Grade 2 (PLT count 75–50) | 4 (17.4) |
| Grade 3 (PLT count 49–25) | 13 (56.5) |
| Grade 4 (PLT count 24–) | 6 (26.1) |
| PLT transfusion ≤ 24 hours, n (%) |  |
| Yes | 11 (47.8) |
| No | 12 (52.2) |
| Local hemostatic treatment, n (%) |  |
| Yes | 21 (91.3) |
| No | 2 (8.7) |
| No. of teeth extracted, median (range) | 2 (1, 5) |
| Extraction diagnosis, n (%) |  |
| Periodontitis | 8 (34.8) |
| Symptomatic/asymptomatic apical periodontitis | 13 (56.5) |
| Symptomatic/asymptomatic pulpitis/fracture | 2 (8.7) |
| Pericoronitis | 0 (0) |
| Other (biopsy, alveoplasty) | 0 (0) |
| Type of dentoalveolar surgery, n (%) |  |
| Non-surgical/surgical extraction 1 tooth (also: biopsy alveoplasty) | 11 (47.8) |
| Non-surgical/surgical extraction  ≥ 2 teeth | 12 (52.2) |
| Adjacent teeth ≥ 2, n (%) |  |
| Yes | 9 (39.1) |
| No | 14 (60.9) |

***PLT*** platelet, ***AML/MDS*** acute myeloid leukemia, myelodysplastic syndrome, ***CTCAE*** Common Terminology Criteria for Adverse Events
^a^including acute lymphoblastic leukemia, chronic lymphocytic leukemia, mantle cell lymphoma, and diffuse large B cell lymphoma

^b^including hemophagocytic lymphohistiocytosis, and aplastic anemia
