# Supplementary material for: Postoperative bleeding after dentoalveolar surgery in patients with thrombocytopenia—are prophylactic platelet transfusions necessary?
Source: Support Care Cancer. 2024 Oct 7;32(10):703. doi: 10.1007/s00520-024-08917-1 (PMC11456549; doi:10.1007/s00520-024-08917-1)
Supplement: Supplementary file 3 — Supplementary file3 (DOCX 2278 KB) [file 520_2024_8917_MOESM3_ESM.docx]

**Postoperative bleeding after dentoalveolar surgery in patients with thrombocytopenia - are prophylactic platelet transfusions necessary?**

Johan Lundström^1,2^, Samuel Wiqvist^3^, Martin Jädersten^4,5^, Victor Tollemar^1^,
Karin Garming Legert^1^

**Affiliations**^1^Department of Dental Medicine, Karolinska Institutet, Stockholm, Sweden
^2^Public Dental Health Service Stockholm AB, Stockholm, Sweden
^3^Department of Learning, Informatics, Management & Ethics (LIME), Karolinska Institutet, Stockholm, Sweden
^4^Department of Hematology, Karolinska University Hospital, Stockholm, Sweden
^5^Center for Hematology and Regenerative Medicine (HERM), Karolinska Institutet, Stockholm, Sweden

Johan Lundström: johan.n.lundstrom@regionstockholm.se

**Supplementary information: Journal notes regarding the seven POB events, the first-time visit, and additional visits.**

NOTE: “Visit 1” is the first-time visit

Patient ID 10

A review of the patient’s hematological journal revealed that the patient was in a general state of cachexia and suffered from kidney failure, which could have been contributing factors to the POB event. Patient 10 had four dental visits.

Visit 1: NPOB was noted. The patient had a pre-transfusion PLT count of 26*10^9^/L. Prophylactic PLT transfusion was given pre-treatment. Two teeth were removed non-surgically and/or surgically (teeth 16, 17).

Visit 2: one POB event was noted. The patient had a pre-transfusion PLT count of 44*10^9^/L. Prophylactic PLT transfusion was given pre-treatment. Two or more teeth were removed non-surgically and/or surgically. The POB event was described as a minor POB event the following day that could be managed with local hemostatic treatment. Three teeth were removed non-surgically or surgically (teeth 25, 26, 27).

Visit 3: NPOB was noted. The patient had a pre-transfusion PLT count of 41*10^9^/L. Prophylactic PLT transfusion was given pre-treatment. One tooth was removed non-surgically or surgically (tooth 21).

Visit 4: NPOB was noted. The patient had a pre-transfusion PLT count of 33*10^9^/L. Prophylactic PLT transfusion was given pre-treatment. Two teeth were removed non-surgically and/or surgically (teeth 46, 47).

Patient ID 11

A review of the patient’s hematological journal revealed no other risk factor that could be considered contributory to the POB event. The patient had a pre-transfusion PLT count of 36*10^9^/L. Prophylactic PLT transfusion was given pre-treatment. Perioperatively, a POB event occurred, and the patient was transferred to the hematological ward for another PLT transfusion and given local hemostatic treatment before the POB event could be managed. Two teeth were removed non-surgically and/or surgically (teeth 16, 47).

Patient ID 18

A review of the patient’s hematological journal revealed a patient with full neutropenia and sepsis due to bacterial sinusitis; these may have influenced the post-operative healing process and could have been contributing factors to the POB event. The patient had a pre-transfusion PLT count of 13*10^9^/L. Prophylactic PLT transfusion was given pre-surgically. A POB event was noted and described as moderate perioperative bleeding that was controlled with local hemostatic treatment. Two teeth were removed non-surgically and/or surgically (teeth 25, 26).

Patient ID 42

A review of the patient’s hematological journal revealed that the patient was neutropenic. During visit 4, when a POB event was noted, the patient also had neutropenic fever, which could have been a contributing factor to the POB event. Patient 42 had five dental visits.

Visit 1: NPOB was noted. The patient had a PLT count of 45*10^9^/L. No prophylactic PLT transfusion was given pre-treatment. Five teeth were removed non-surgically and/or surgically (teeth 11, 12, 13, 14, 15).

Visit 2: NPOB was noted. The patient had a PLT count of 45*10^9^/L. No prophylactic PLT transfusion was given pre-treatment. Three teeth were removed non-surgically and/or surgically (teeth 21,24, 25).

Visit 3: NPOB was noted. The patient had a PLT count of 64*10^9^/L. No prophylactic PLT transfusion was given pre-treatment. Four teeth were removed non-surgically and/or surgically (teeth 22, 23, 27, 28).

Visit 4: One POB event was noted. The patient had a PLT count of 17*10^9^/L. No prophylactic PLT transfusion was given pre-treatment. The POB event was described as moderate bleeding perioperatively, which could be managed with local hemostatic treatment. Three teeth were removed non-surgically and/or surgically (teeth 46, 47, 48).

Visit 5: NPOB was noted. The patient had a PLT count of 38*10^9^/L. No prophylactic PLT transfusion was given pre-treatment. Two teeth were removed non-surgically and/or surgically (teeth 37, 38).

Patient ID 49

A review of the patient’s hematological journal revealed that the dentoalveolar surgery was performed on day 5 of an intense course of cytostatic treatment (FA-IDA) for acute myeloid leukemia (AML). This treatment affects the healing process and could have been a contributing factor to the POB event. The patient had a PLT count of 60*10^9^/L. No prophylactic PLT transfusion was given pre-treatment. A POB event was noted and described as POB that continued for days following dental treatment but was controlled with local hemostatic treatment. Three teeth were removed non-surgically and/or surgically (teeth 17, 27, 47).

Patient ID 61

A review of the patient’s hematological journal revealed that the patient had an ongoing systemic infection during dental treatment that could have been a contributing factor to the POB event. Patient 61 had two dental visits.

Visit 1: a POB event was noted. The patient had a PLT count of 19*10^9^/L. No prophylactic PLT transfusion was given pre-treatment. The POB event was noted perioperatively and described as intense bleeding that needed both local hemostatic treatment and prophylactic PLT transfusion in order to be managed. Three teeth were removed non-surgically and/or surgically (teeth 26, 27, 28).

Visit 2: NPOB event was noted. The patient had a pre-transfusion PLT count of 11*10^9^/L. Prophylactic PLT transfusion was given pre-treatment. One tooth was removed non-surgically and/or surgically (tooth 38).

Patient ID 72

A review of the patient’s hematological journal revealed administration of high doses of cortisone but no obvious contributing factor to the POB event. The patient had a PLT count of 62*10^9^/L. No prophylactic PLT transfusion was given pre-treatment. The POB event was noted postoperatively, was described as moderate bleeding at the hematology ward, and was controlled with local hemostatic treatment. One tooth was removed non-surgically and/or surgically (tooth 38).
